# Supplementary material for: Near-infrared manipulation of multiple neuronal populations via trichromatic upconversion
Source: Nat Commun. 2021 Sep 27;12:5662. doi: 10.1038/s41467-021-25993-7 (PMC8476604; doi:10.1038/s41467-021-25993-7)
Supplement: Supplementary file 1 — Supplementary Information [file 41467_2021_25993_MOESM1_ESM.pdf]

Supplementary Information

Xuan Liu, Heming Chen, Yiting Wang, Yueguang Si, Hongxin Zhang, Xiaomin Li*, Zhengcheng Zhang, Biao Yan, Su Jiang, Fei Wang, Shijun Weng, Wendong Xu, Dongyuan Zhao, Jiayi Zhang*, Fan Zhang*

Contents

I. Supplementary Methods 2

II. Supplementary Figures 5

Supplementary Note 1: Fabrication and characterization of the trichromatic UCNPs 6

Supplementary Note 2: Single particle imaging and in situ luminescence 8

Supplementary Note 3: Effect of sensitizer concentration tuning. 14

Supplementary Note 4: Effect of shell thickness tuning. 17

Supplementary Note 5: Comparison of involved three dissipation process. 18

Supplementary Note 6: Energy dissipation process of 808 nm with Nd^{3+} as sensitizer. 20

Supplementary Note 7: Effect of the same sensitizer in both core and shell. 21

Supplementary Note 8:Coupling cross-relaxation and surface passivation for pure red emission. 24

Supplementary Note 9:Interpretation of the energy dissipation mechanism. 26

Supplementary Note 10: Interpretation of the change of sensitizer lifetime and de-saturation of sensitizer. 26

Supplementary Note 11:NIR penetration test and upconversion efficiency 37

Supplementary Note 12: NIR thermal effect induced heat distribution between brain surface and local tissue. 39

I. Supplementary Methods

Synthesis of trichromatic upconversion nanoparticles

Materials. Gadolinium (III) chloride anhydrous ($GdCl_3$, 99.99 %), ytterbium (III) chloride anhydrous ($YbCl_3$, 99.9 %), yttrium(III) chloride anhydrous (YCl_3, 99.9 %), erbium (III) chloride anhydrous ($ErCl_3$, 99.9 %), thulium (III) chloride anhydrous ($TmCl_3$, 99.9 %), sodium trifluoroacetate (Na-TFA, 98 %), 1-octadecene (ODE, 90 %), oleic acid (OA, 90 %) were purchased from Sigma-Aldrich. Sodium hydroxide (NaOH, 96 %), ammonium fluoride (NH_4F, 96 %) were obtained from Beijing Chemical Reagents Co. Ltd. Polydimethylsiloxane (PDMS) base and curing agent were purchased from DOW Corning Co.Ltd. AAV-hChR2, AAV-C1V1, AAV-ChrimonsonR viruses were obtained from Shanghai Taitool Bioscience Co. Ltd. All chemicals were used as received without any further purification.

Preparation of shell precursors. Y-OA (0.10 M) precursor: A mixture of YCl_3 (2.50 mmol), OA (10.00 mL), and ODE (15.00 mL) was loaded in a reaction container and heated at 140 °C under vacuum with magnetic stirring for 30 min to remove residual water and oxygen. Then, the colorless Y-OA precursor solution (0.10 M) was obtained.
Gd-OA(0.10 M), Er-OA(0.10 M), Yb-OA(0.10 M), Nd-OA(0.10 M), Tm-OA(0.10 M) precursors: The synthesis of the other precursors were carried out all the same as that of the Y-OA precursor except 2.50 mmol of $GdCl_3$, 2.50 mmol of $ErCl_3$, 2.50 mmol of $YbCl_3$, 2.50 mmol of $NdCl_3$, 2.50 mmol of $TmCl_3$ were used instead of 2.50 mmol of YCl_3. Na-TFA-OA (0.40 M) precursor: A mixture of Na-TFA (4.00 mmol) and OA (10.00 mL) was loaded in a container at room temperature under vacuum with magnetic stirring to remove residual water and oxygen. Then the colorless Na-TFA-OA precursor solution (0.40 M) was obtained.

Synthesis of β-$NaErF_4$@$NaYF_4$@$NaGdF_4$:20%Yb,2%Er@$NaGdF_4$:20%Yb@ $NaGdF_4$:50%Nd,10%Yb@$NaYF_4$@$NaGdF_4$:50%Yb,1%Tm@$NaYF_4$.
Note: The construction of trichromatic upconversion nanoparticle required multistep engineering. Each step must be repeat several times to completely block ion diffusion and surface exchange during synthesis.
Synthesis of β-$NaErF_4$ core nanoparticles. The synthesis of β-$NaErF_4$ core nanoparticles with the size of ~ 22 nm were similar to the previously reported thermolysis method[1]. $ErCl_3$ (1.00 mmol), OA (6.00 mL) and ODE (15.00 mL) were mixed together and heated to 140 °C under vacuum until formed a clear solution, after that, the solution was cooled down to room temperature. A solution of NaOH (2.50 mmol) and NH_4F (4.00 mmol) in methanol (10.00 mL) was added. The resultant mixture was stirred for half an hour. The mixture was then heated to 70 °C and maintained for half an hour to remove the methanol. Afterward, the solution was heated to 290 °C and maintained for 100 min under

a gentle argon flow. Then, the solution was cool down to room temperature. The nanoparticles were centrifuged and washed twice with ethanol and finally dispersed in 10 mL of cyclohexane for further use.

Synthesis of β-$NaErF_4$@$NaYF_4$ Core-shell nanoparticles. The fabrication of core-shell nanoparticles were carried out by the one-pot successive layer-by-layer (SLBL) protocol, which was developed by our group previously[2]. The shell precursor was firstly obtained from mixing the Y-OA (0.10 M, 1.00 mL) and Na-TFA-OA (0.40 M, 0.50 mL). Then, 2.50 mL of the purified $NaErF_4$ core nanoparticles cyclohexane solution was mixed with 4.00 mL of OA and 6.00 mL of ODE. The flask was pumped down at 100 °C for 30 min to remove cyclohexane and any residual air. Subsequently, the system was switched to Ar flow and further heated to 270 °C at a rate of ~ 20 °C/min. Then the shell precursors were introduced by dropwise addition at 270 °C with the rate of 3.00 mL/h. The shell thickness can be well tuned by changing the amount of the shell precursors. After injection, the reaction was kept 30 min for ripening. Finally, the obtained $NaErF_4$/$NaYF_4$ (C/S_1) nanoparticles were precipitated and washed several times with ethanol and re-dispersed in cyclohexane for further use. The above coating step was repeated 4 times to make sure a sufficient thickness with complete $NaYF_4$ at surface. Otherwise diffusion and ion exchange during synthesis process would leak the inner erbium ion into outer layers and even very few erbium leakage would lead to cross-response under 980 nm excitation.

Synthesis of β-$NaErF_4$@$NaYF_4$@$NaGdF_4$:20%Yb,2%Er ($CS_{1\text{-}2}$) nanoparticles. The fabrication of β-$NaErF_4$@$NaYF_4$@$NaGdF_4$:20%Yb,2%Er ($CS_{1\text{-}2}$) nanoparticles were similar to aforementioned core-shell nanoparticles. The shell precursor was firstly obtained from mixing the Gd-OA (0.1 M, 5.85 mL), Yb-OA (0.1 M, 1.5 mL), Er-OA (0.1 M, 0.15 mL) and Na-TFA-OA (0.40 M, 3.75 mL). All of the purified $NaErF_4$@$NaYF_4$ (CS_1) nanoparticles were mixed with 4.00 mL of OA and 6.00 mL of ODE. The flask was pumped down at 100 °C for 30 min to remove cyclohexane and residual air. Subsequently, the system was switched to Ar flow and further heated to 270 °C at a rate of ~ 20 °C/min. Then the shell precursors were introduced by dropwise addition at 270 °C with the rate of 3.00 mL/h. The reaction were kept 30 min after injection for ripening. The final product were precipitated and washed several times with ethanol and re-dispersed in cyclohexane for further use.

Synthesis of β-$NaErF_4$@$NaYF_4$@$NaGdF_4$:20%Yb,2%Er@$NaGdF_4$:20%Yb ($CS_{1\text{-}3}$) nanoparticles. The process for coating $NaGdF_4$:20%Yb shell3 was the same as the aforementioned synthesis of β-$NaErF_4$@$NaYF_4$@$NaGdF_4$:20%Yb,2%Er ($CS_{1\text{-}2}$) nanoparticles. But the seed particles were changed to β-$NaErF_4$@$NaYF_4$@$NaGdF_4$:20%Yb,2%Er and shell precursors were changed to Gd-OA (0.10 M, 2.00 mL), Yb-OA (0.10 M, 0.50 mL) and Na-TFA-OA (0.40 M, 1.25 mL).

Synthesis of β-$NaErF_4$@$NaYF_4$@$NaGdF_4$:20%Yb,2%Er@$NaGdF_4$:20%Yb@

$NaGdF_4$:50%Nd,10%Yb ($CS_{1\text{-}4}$) nanoparticles. The process for coating $NaGdF_4$:50%Nd,10%Yb shell4 was the same as the aforementioned synthesis of β-$NaErF_4$@$NaYF_4$@$NaGdF_4$:20%Yb,2%Er ($CS_{1\text{-}2}$) nanoparticles. But the seed particles were changed to β-$NaErF_4$@$NaYF_4$@$NaGdF_4$:20%Yb,2%Er@$NaGdF_4$:20%Yb and shell precursors were changed to Gd-OA (0.05 M, 8.00 mL), Yb-OA (0.05 M, 2.00 mL), Nd-OA (0.05 M, 10.00 mL) and Na-TFA-OA (0.20 M, 10.00 mL).

Synthesis of β-$NaErF_4$@$NaYF_4$@$NaGdF_4$:20%Yb,2%Er@$NaGdF_4$:20%Yb@ $NaGdF_4$:50%Nd,10%Yb@$NaYF_4$ ($CS_{1\text{-}5}$) nanoparticles. The process for coating $NaYF_4$ shell5 was the same as the aforementioned synthesis of β-$NaErF_4$ @$NaYF_4$@$NaGdF_4$:20%Yb,2%Er ($CS_{1\text{-}2}$) nanoparticles. But seed particles were changed to β-$NaErF_4$@$NaYF_4$@$NaGdF_4$:20%Yb,2%Er@$NaGdF_4$:20%Yb@ $NaGdF_4$:50%Nd,10%Yb and shell precursors were changed to Y-OA (0.10 M, 10.00 mL) and Na-TFA-OA (0.40 M, 5.00 mL). The above coating step was repeated twice to make sure a sufficient thickness with complete $NaYF_4$ at surface preventing from diffusion of Nd^{3+} into outer layers.

Synthesis of β-$NaErF_4$@$NaYF_4$@$NaGdF_4$:20%Yb,2%Er@$NaGdF_4$:20%Yb@ $NaGdF_4$:50%Nd,10%Yb@$NaYF_4$@$NaGdF_4$:80%Yb,1%Tm ($CS_{1\text{-}6}$) nanoparticles. The process for coating $NaGdF_4$:80%Yb,1%Tm shell6 was the same as the aforementioned synthesis of β-$NaErF_4$@$NaYF_4$@$NaGdF_4$:20%Yb,2%Er ($CS_{1\text{-}2}$) nanoparticles. But the seed particles were changed to β-$NaErF_4$@$NaYF_4$@$NaGdF_4$:20%Yb,2%Er@$NaGdF_4$:20%Yb@$NaGdF_4$:50%Nd,10%Yb@$NaYF_4$ and shell precursors were changed to Gd-OA (0.10 M, 1.50 mL), Yb-OA (0.10 M, 6.00 mL), Tm-OA (0.10 M, 75 μL) and Na-TFA-OA (0.40 M, 3.75 mL). This coating step of shell6 was repeated 4 times to make sure a sufficient shell thickness to dissipate 980 nm excitation energy.

Synthesis of β-$NaErF_4$@$NaYF_4$@$NaGdF_4$:20%Yb,2%Er@$NaGdF_4$:20%Yb@ $NaGdF_4$:50%Nd,10%Yb@$NaYF_4$@$NaGdF_4$:80%Yb,1%Tm@$NaYF_4$ ($CS_{1\text{-}7}$) nanoparticles. The process for coating of $NaYF_4$ shell7 was the same as the above mentioned synthesis of β-$NaErF_4$@$NaYF_4$@$NaGdF_4$:20%Yb,2%Er ($CS_{1\text{-}2}$) nanoparticles. But the seed particles were changed to β-$NaErF_4$@$NaYF_4$@$NaGdF_4$:20%Yb,2%Er@$NaGdF_4$:20%Yb@$NaGdF_4$:50%Nd,10%Yb@$NaYF_4$@N aGdF4:80%Yb,1%Tm and shell precursors were changed to Y-OA (0.1 0M, 10.00 mL) and Na-TFA-OA (0.40 M, 5.00 mL).

II. Supplementary Figures

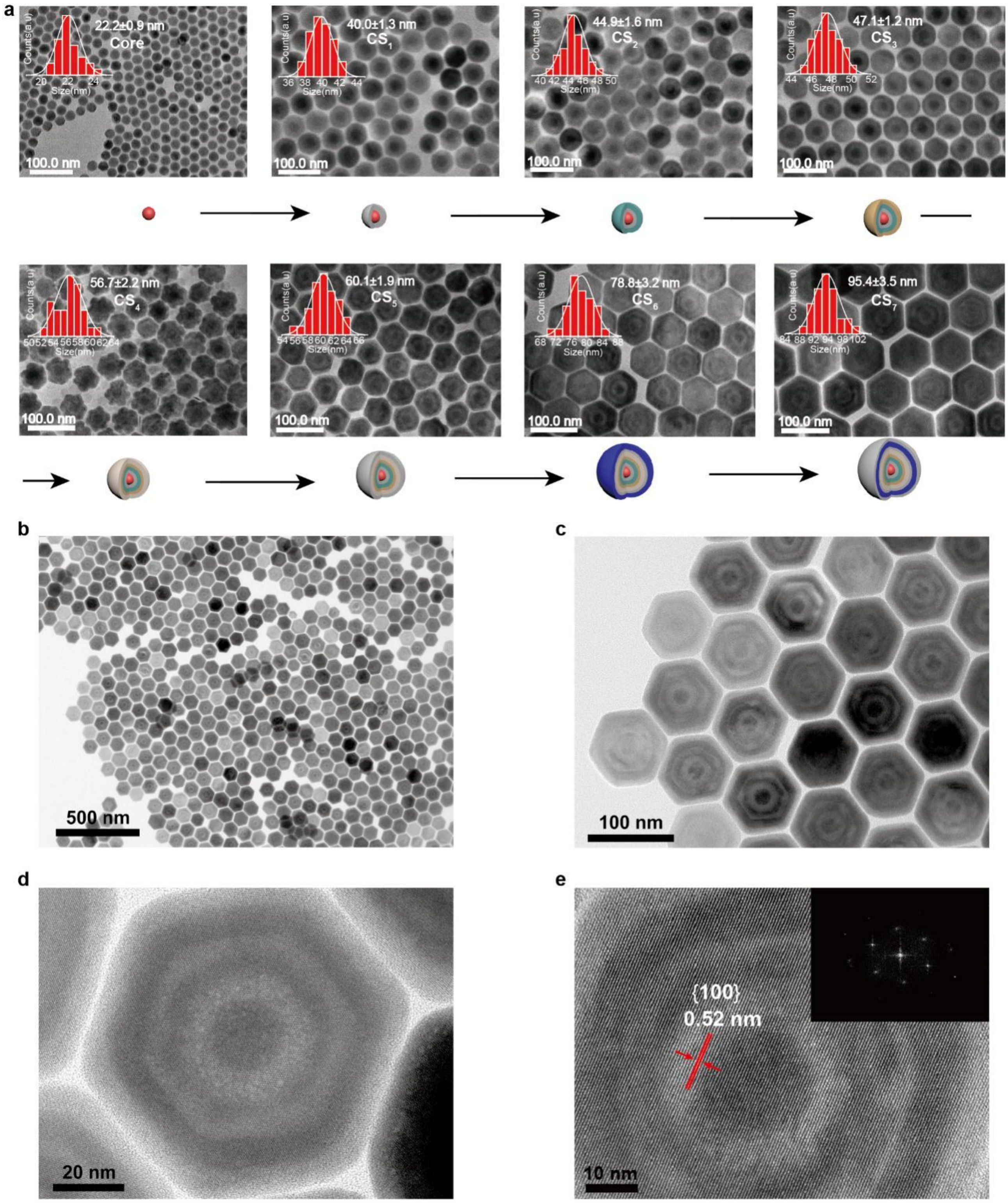

Supplementary Fig. 1. **Fabrication process and the corresponding characterization of the trichromatic UCNPs. a**, Transmission electron microscope (TEM) images of the as-synthesized UCNPs after each extending step for fabricating core-multishell structured

$NaErF_4@NaYF_4@NaGdF_4$:20%Yb,2%Er@$NaGdF_4$:20%Yb@$NaGdF_4$:50%Nd,10%Yb@$NaYF_4$@$NaGdF_4$:80%Yb,1%Tm@$NaYF_4$ UCNPs. Size distribution histograms with mean diameter and standard deviation were obtained from the statistical result of more than 50 particles. The average diameter of $NaErF_4$ core was 22.2 nm, which gradually increased to 40.0, 44.9, 47.1, 56.7, 60.1, 78.8 and 95.4 nm after extending each shell. The thickness of shells was tuned by adjusting the amount of precursors introduced in each step. **b-e**, High resolution transmission electron microscope images along with corresponding fast Fourier transform of as-synthesized multi-layer UCNPs. The synthesis was repeated for 4 times with similar results.

Supplementary Note 1: Fabrication and characterization of the trichromatic UCNPs

The obtained UCNPs show uniform morphology, discernible contrast for core-multishell nanostructure and crystalline hexagonal phase without any significant impurity. Layer by layer epitaxial growth strategy was used for construction of the core-shell structured UNCPs. Fabrication process repeat at least twice in construction of each layer, which aimed at blocking the diffusion of ions between layers as well as for a sufficient dissipation shell thickness. The seemingly heterogeneous growth of CS4 is originated from large seed/precursor ratio and the misfit strain in core-shell construction which do not affect the intrinsic optical property[4].

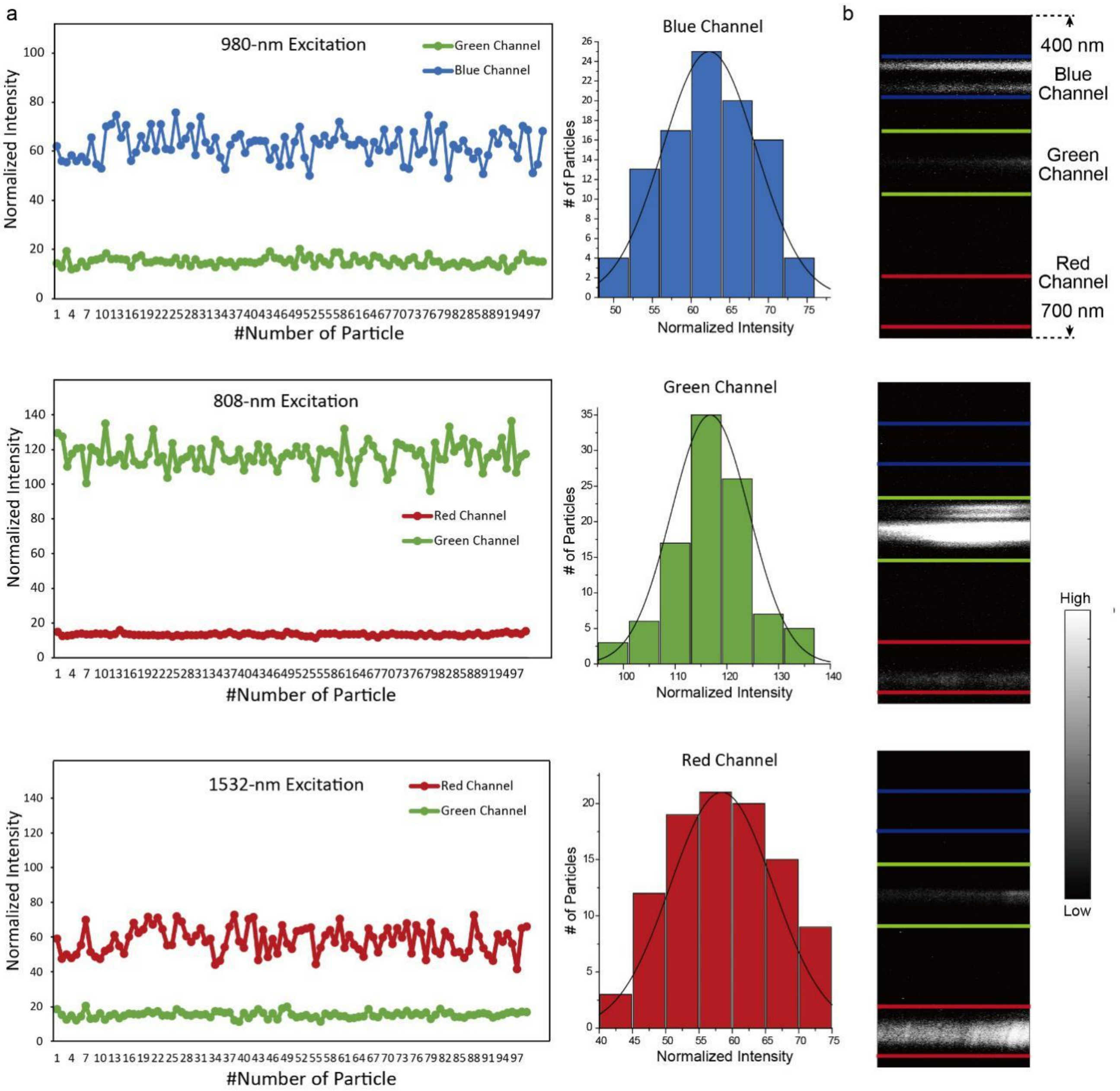

Supplementary Fig. 2. Information of color channel intensity of individual spot. a, Original data of color channel intensity from each spot and histogram of dominant bands intensity under 980 nm, 808 nm and 1532 nm excitation. **b,** Example of scanning spectra data from single spots. Channel range: Blue 438 nm-480 nm, Green 507 nm-560 nm, Red 641 nm-688 nm. Single particle spots were first searched on a cleansed coverslip dispersed with dilute trichromatic UCNPs solution under a max laser power density about ~500 W/cm^{-2}. Laser is then switched to low power density of ~180 mW/mm^{-2} for collecting luminescence images and scanning spectra ranging from 400 nm to 700 nm. Source data are provided as a Source Data file.

Supplementary Note 2: Single particle imaging and in situ luminescence

Images were obtained in a darkroom with up to 60 seconds exposure time. Channel intensity of three color were derived from a calculation of average integral intensity of all spot scanning spectra images. Histograms of each color channel intensity from over 100 luminescent spots show well fitted Gaussian distribution. The non-steep intensity curve suggest that these results are most from single UCNPs.

a

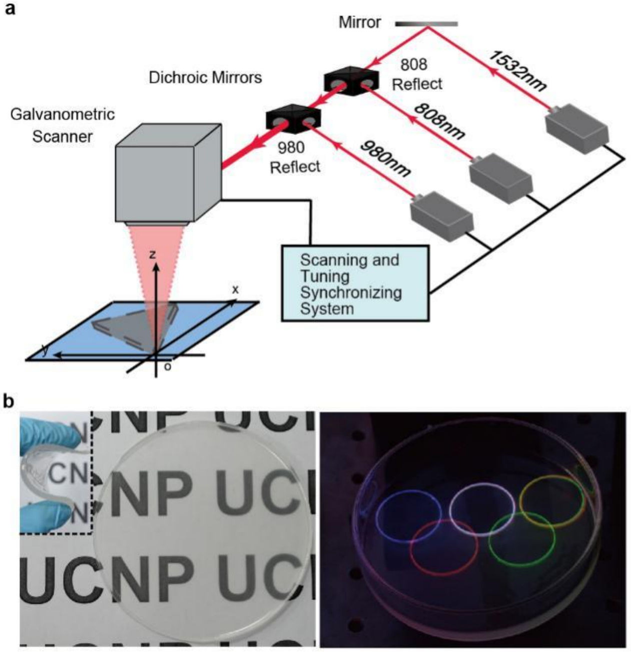

b

c

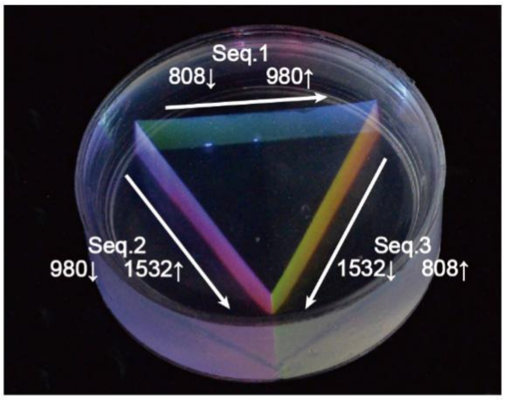

d

	Setting of output power change (W)		
	980 nm	808 nm	1532 nm
Sequence 1	0 → 5	2 → 0	-
Sequence 2	5 → 0	-	0 → 3.5
Sequence 3	-	0 → 2	3.5 → 0

*Scantime = 10 s × 3 Spot size < 1 mm^2

Supplementary Fig. 3. **Scheme of the synchronous scanning process**. **a**, Schematic illustration of the synchronous galvanometric scanning system equipped with three NIR continuous-wave lasers for dynamic tuning. **b**, Photographs of obtained flexible polydimethylsiloxane (PDMS) monolith infused with the trichromatic UCNPs and the Olympic rings multi-color pattern under the excitations of near-infrared (NIR) continuous-wave (CW) lasers. Image is obtained via synchronous scanning and laser power tuning. **c**, Luminescent triangular prism in nanocrystal/PDMS substrate at the excitations of focus NIR lights with gradient power. Image is obtained via synchronous scanning and laser power tuning system. Sequence arrows indicate a continuous power change of excitation lasers. **d**, Table of the program setting of lasers output power adjustment in **c**. See methods for details.

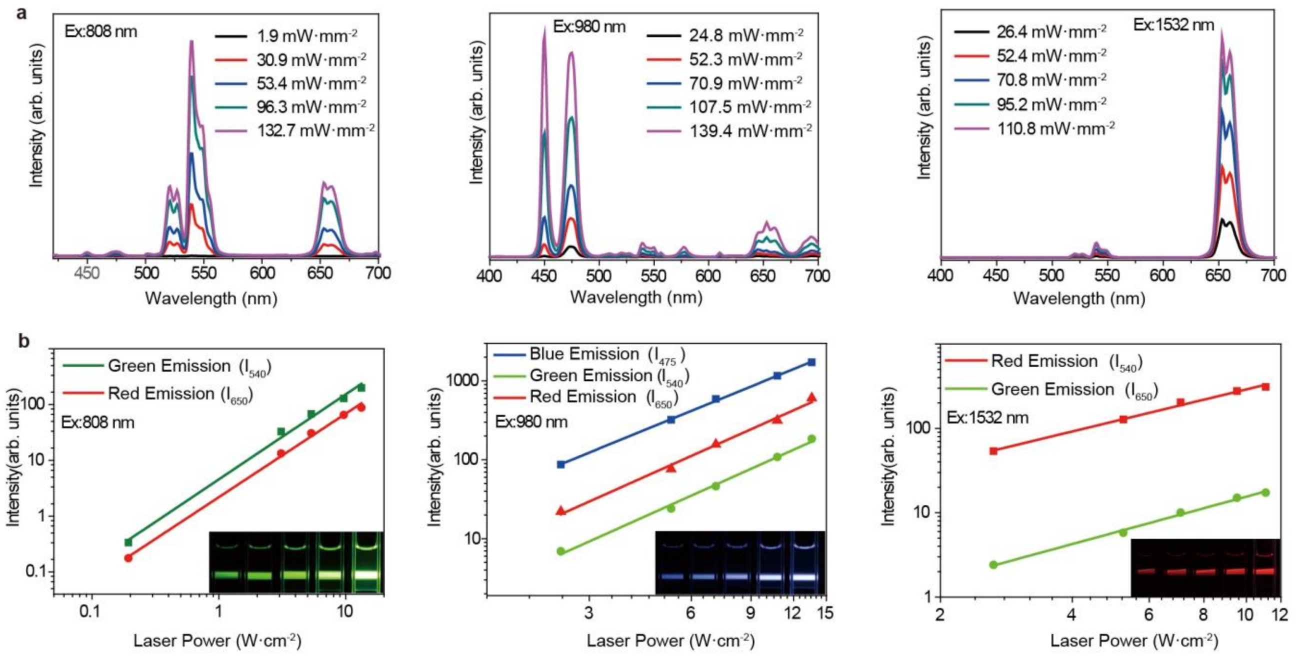

Supplementary Fig. 4. **Excitation Power density dependent upconversion emission of the trichromatic UCNPs. a**, Upconversion luminescence spectra under different excitation power density. **b**, Corresponding intensity integration of the characteristic emission peaks (Insert: photographs of UCNPs solution under different excitation power density). Logarithmic plots of emission intensity versus excitation power density (from ~20 to ~130 mW/mm^2) show nearly parallel with each other, indicating the relative intensity among blue (475 nm), green (540 nm) and red (640 nm) emissions remains approximately constant. The trichromatic emission of as-synthesized UCNPs is excitation power independent. The upper threshold estimated from data is over ~ 120 mW/mm^2. Source data are provided as a Source Data file.

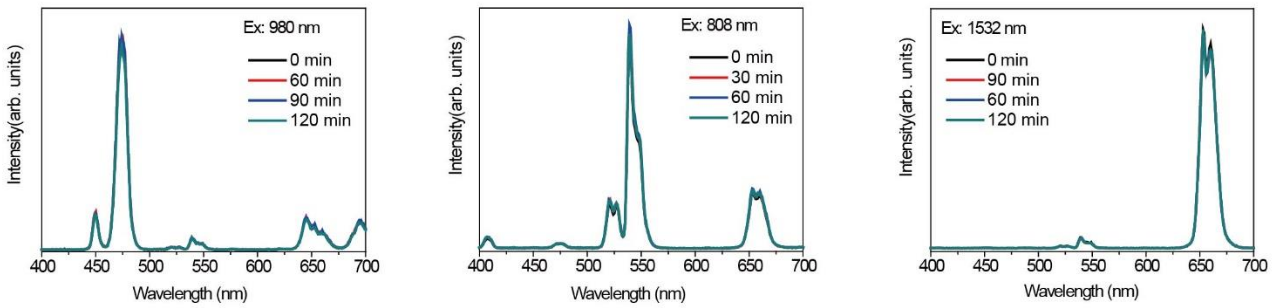

Supplementary Fig. 5. **Excitation photostability of trichromatic upconversion emission. a-c**, Luminescence spectra of the trichromatic UCNPs under excitations of 980, 808 and 1532 nm with ~ 50 mW/mm^{-2} power density for 0-120 min. The upconversion emissions show good luminescent stability and reproducibility without noticeable emission intensity change under laser excitation up to 120 min. Source data are provided as a Source Data file.

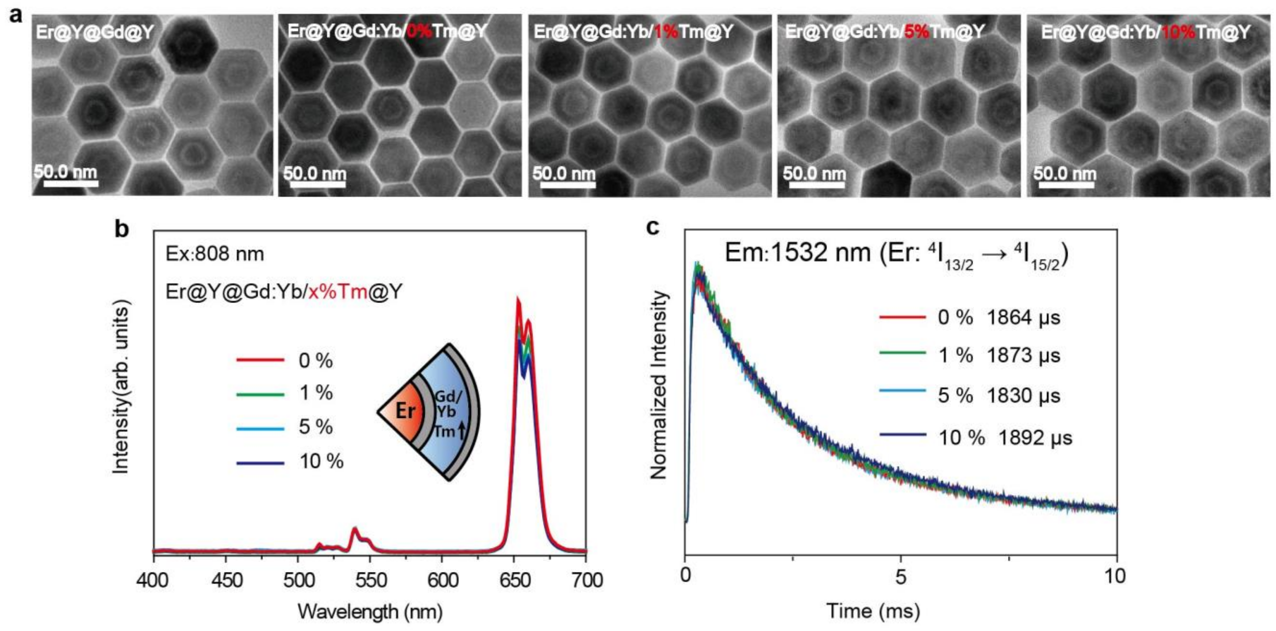

Supplementary Fig. 6. **Activators amount based energy dissipation process. a,** Transmission electron microscope (TEM) images of obtained $NaErF_4@NaYF_4@NaGdF_4$:Yb/Tm@$NaYF_4$ UCNPs with different Tm^{3+} doping concentration in $NaGdF_4$:Yb/Tm layer and $NaErF_4@NaYF_4@NaGdF_4$ @$NaYF_4$ UCNPs. The synthesis was repeated twice with similar results. **b**, Upconversion emissions spectra under 808 nm of $NaErF_4@NaYF_4@$ $NaGdF_4$:49%Yb/x%Tm@$NaYF_4$ UCNPs (x = 0, 1, 5, 10). Shell thickness is around 4 nm. **c**, Luminescence decay curves of the obtained UCNPs in (**a)** at 1532 nm under 980 nm excitation. UCNPs were dispersed in cyclohexane for collection of upconversion emission spectra with excitations of 980 nm at a power density of ~ 45 mW/mm^2. Source data are provided as a Source Data file.

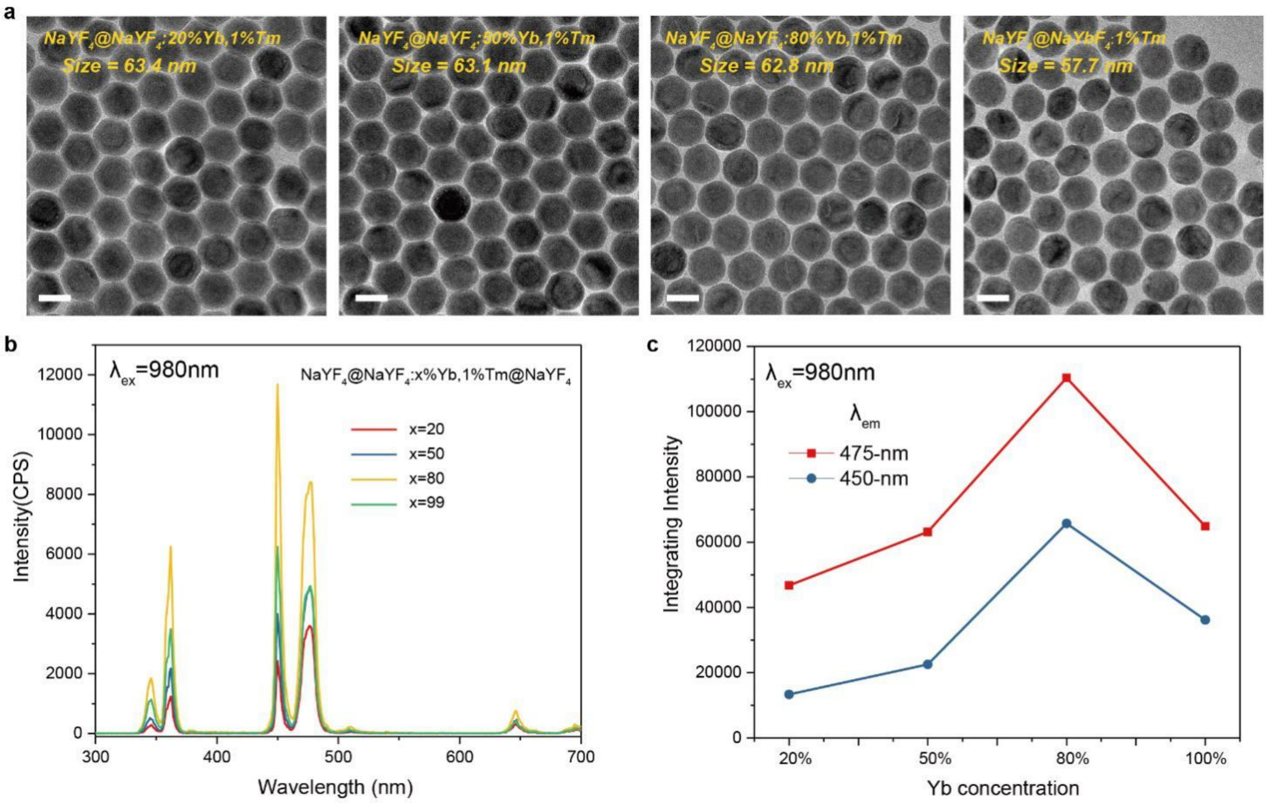

Supplementary Fig. 7 Optimizing Tm^{3+} by sensitizer concentration. a, TEM images of UCNPs $NaYF_4@NaYF_4$: x%Yb,1%Tm, which derived from the same core. Scale bar=50 nm. The synthesis was repeated twice with similar results. **b**, Luminescence spectra of UCNPs $NaYF_4@NaYF_4$: x%Yb,1%1Tm@$NaYF_4$ (x= 20, 50, 80 99). **c,** Integrating Intensity of 450 nm and 475 nm bands as a function of Yb doping concentration in **b**. Source data are provided as a Source Data file.

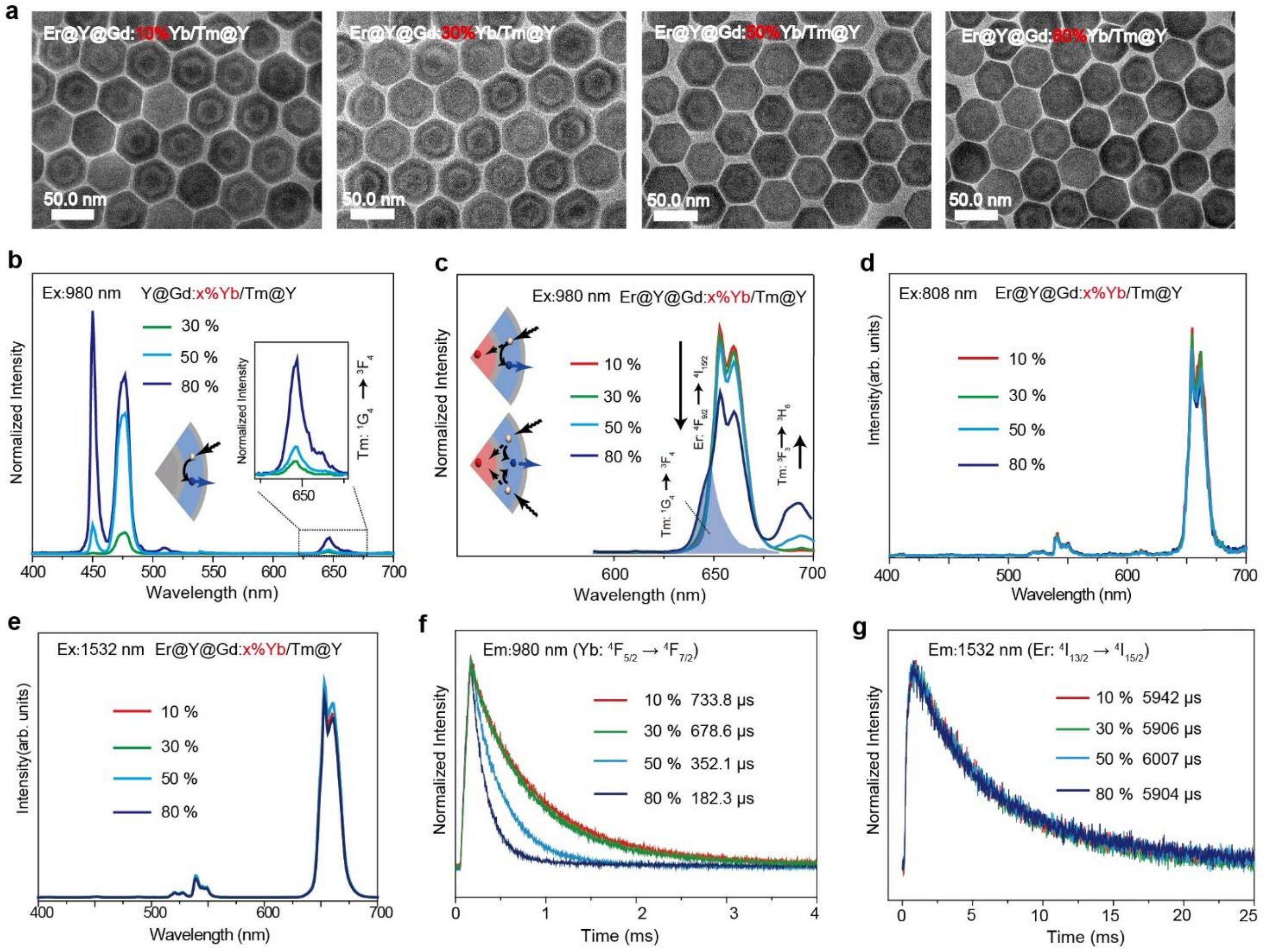

Supplementary Fig. 8. **Sensitizer amount based absorption process. a**, Transmission electron microscope (TEM) images of obtained $NaErF_4$@$NaYF_4$@$NaGdF_4$:x%Yb/1%Tm@$NaYF_4$ UCNPs with different Yb^{3+} doping amount in $NaGdF_4$:Yb/Tm layer. The synthesis was repeated twice with similar results. **b**, Upconversion emission spectra of $NaYF_4$@$NaGdF_4$:x%Yb/1%Tm@$NaYF_4$ UCNPs under 980 nm excitation with different Yb^{3+} doping amount. **c-e**, Upconversion emission spectra under 980,808 and 1532 nm excitation. Emission intensity is normalized by UCNPs with pure matrix $NaGdF_4$ in S2. **f-h**, Luminescence decay curves of the obtained UCNPs at 980 and 1532 nm under 980 nm excitation. UCNPs were dispersed in cyclohexane for collection of upconversion emission spectra under 980 and 808 nm excitation at a power density of ~ 30 mW/mm^2. Source data are provided as a Source Data file.

Supplementary Note 3: Effect of sensitizer concentration tuning.

When increasing Yb^{3+} concentration to 80% with Tm^{3+} doping, the excited state transition $^1G_4 \rightarrow {}^3F_4$ of Tm^{3+} related to emission peaked at 645 nm is enhanced (**Supplementary Fig. 8b**). This band would mix with the emission band of Er^{3+} peaked at 651 nm even under a low excitation power density, which

limit the quantitative comparison of integral strength of inner emission. Although the sample with 80% Yb^{3+} indeed performs the lowest red emission intensity, given that a comparable parameter can better quantify the effect, we chose 49% of Yb^{3+} as typical example and then investigate the factor of Tm^{3+} doping and shell thickness without the influence of Tm^{3+} emission.

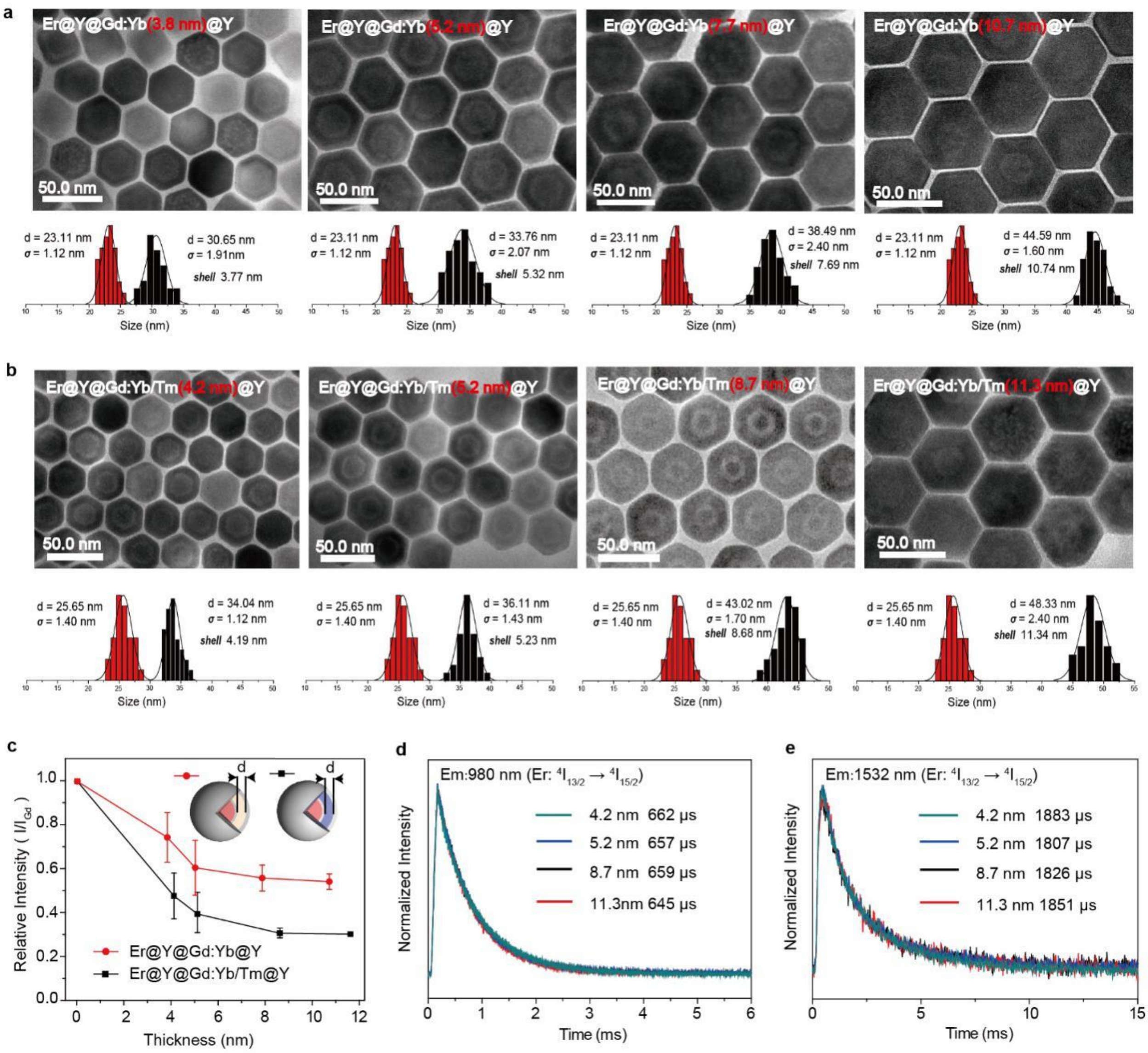

Supplementary Fig. 9. **Shell Thickness based filtration process. a-b**, Transmission electron microscope (TEM) images of the obtained core-shell structured $NaErF_4$@$NaYF_4$@$NaGdF_4$:Yb@$NaYF_4$ and $NaErF_4$@$NaYF_4$@$NaGdF_4$:Yb/Tm@$NaYF_4$ (C/S1/S2/S3) UCNPs with different thickness of shell S2. Histograms of size distribution for core and core/shell nanoparticles are shown corresponding to the TEM images in (a) and (b). **c,** Dissipation shell thickness dependent relative intensity plot of red emission of $NaErF_4$@$NaYF_4$@$NaGdF_4$:49%Yb@$NaYF_4$ and $NaErF_4$@$NaYF_4$@$NaGdF_4$:49%Yb/1%Tm@$NaYF_4$ UCNPs. Emission intensity is normalized by intensity from UCNPs with shell component of pure $NaGdF_4$ matrix in Shell2. n = 3 independent synthesized samples. Data are presented as mean values +/- SEM of these samples. **d-e**, Luminescence decay curves of the 980 and 1532 nm emissions of $NaErF_4$@$NaYF_4$@$NaGdF_4$:Yb/Tm@$NaYF_4$ UCNPs under 980 nm laser excitation. Source data are provided as a Source Data file.

Supplementary Note 4: Effect of shell thickness tuning.

The thickness of the dissipation shell (S2) is well tuned from ~ 4 to ~ 11 nm in $NaErF_4$@$NaYF_4$@$NaGdF_4$:50%Yb@$NaYF_4$ and $NaErF_4$@$NaYF_4$@$NaGdF_4$:50%Yb/1%Tm@$NaYF_4$ UCNPs. Inner core's emission intensity decreased with the increase of S2. While there is no significance difference in the luminescence lifetime at either 980 nm of Yb^{3+} or 1532 nm of the inner core's emission for UCNPs with a $NaGdF_4$:50%Yb,1%Tm dissipation shell.

a

Case	Excitation Wavelength	Related layer(s)	Sensitizer		Absorption Cross-Section
#1	980-nm	Outer Part<br>Middle Part	Yb	Yb	σ(Yb)= σ(Yb)
#2	980-nm	Core<br>Outer Part	Er	Yb	σ(Yb)> σ(Er)
#3	808-nm	Core<br>Middle Part	Er	Nd	σ(Nd)> σ(Er)

b

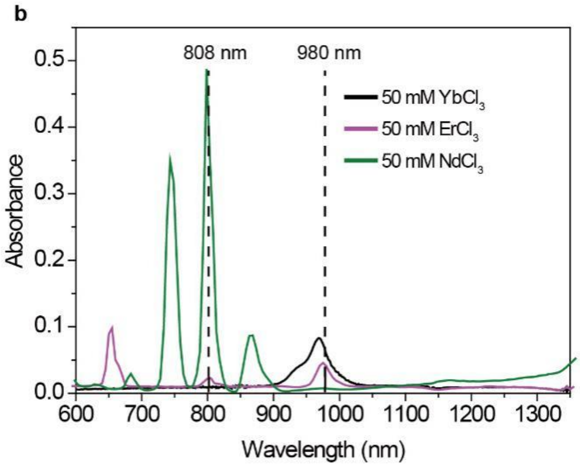

Supplementary Fig. 10. **Comparison of three dissipation process involved in the trichromatic UCNPs. a**, Table summarizing the three cases involved in trichromatic dissipation process. **b**, The extinction spectra of lanthanide Yb/Er/Nd in chloride aqueous solutions with the same concentration. Source data are provided as a Source Data file.

Supplementary Note 5: Comparison of involved three dissipation process.

Three cases of dissipation process are involved in trichromatic UCNPs. Case #1 discussed in previous reports is corresponding to blue/green emission color, which is related to the same sensitizer Yb. The responsive emission is mainly derived from higher amount of Yb^{3+} in outer shell gaining stronger absorption.

Case #2 discussed in the main text is corresponding to blue/red emission which shows that with larger absorption cross-section of Yb than Er at 980 nm, enhancing energy consumption will be much effective. We can deduce the transmitted ratio from the change of inner emission intensity with or without dissipation shell. The relationship of excitation power and inner emission intensity ratio is:

$$\frac{I_1}{I_0} = (\frac{P_1}{P_0})^n$$

where I_1, I_0 are inner emission intensity with or without dissipation shell, P_1, P_0 are the excitation power density with or without dissipation shell. For ~651 nm red emission band from Er, the number of sensitizing steps n is ~2 (CR involved). From experimental results, a shell structure of 49%Yb1%Tm will give 56.4% emission suppression, which means 66% (P_1/P_0) of excitation intensity was left after penetration. Similarly, a structure of 80%Yb1%Tm gives 60% intensity left.

Case #3 discussed in Supplementary Figure 11 is corresponding to red/green emission, which is related to sensitizer Nd and Er. Because the absorption cross-section of Nd is much larger than Er at 808 nm, the dissipation efficiency of middle part is much higher than other process.

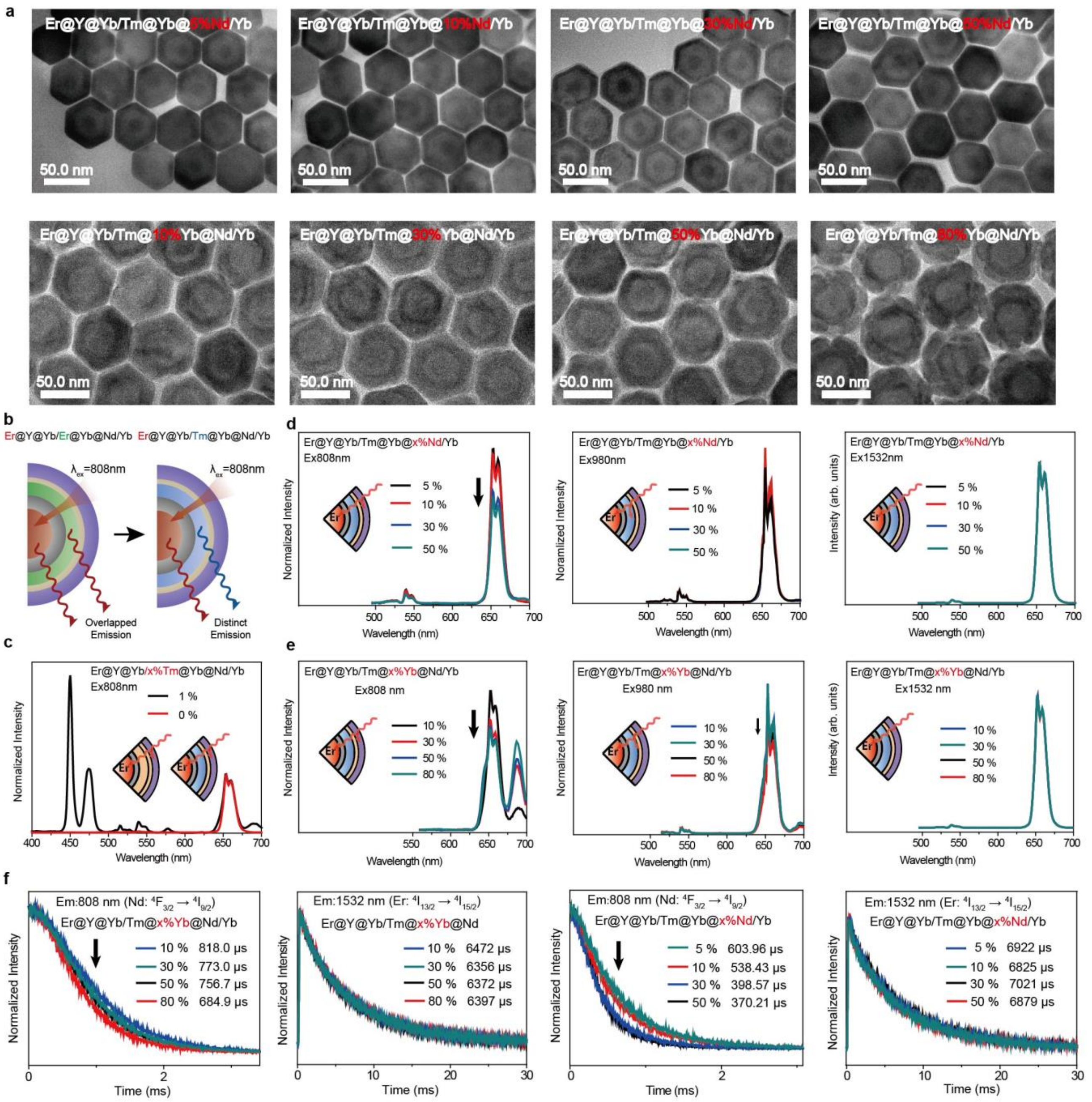

Supplementary Fig. 11. Energy dissipation process of 808 nm with Nd^{3+} as sensitizer. a, TEM images of $NaErF_4$@$NaYF_4$@$NaGdF_4$:Yb/Tm@$NaGdF_4$:Yb@$NaGdF_4$:Yb/Nd UCNPs with different doping amount of Nd^{3+} in $NaGdF_4$:Yb/Nd shell or Yb^{3+} in $NaGdF_4$:Yb shell. **b**, Structure illustration of core and middle part of trichromatic UCNPs with Er or Tm as emissive center. **c**, Luminescence spectra under excitations of 808 nm of $NaErF_4$@$NaYF_4$@$NaGdF_4$:Yb/(Tm)@$NaGdF_4$:Yb@$NaGdF_4$:Yb/Nd UCNPs with or without Tm doping in shell2. The synthesis was repeated twice with similar results. **d**, Luminescence spectra at ~ 650 nm under excitations of 808, 980 and 1532 nm of $NaErF_4$@$NaYF_4$@$NaGdF_4$:Yb/Tm@$NaGdF_4$:Yb@$NaGdF_4$:Yb/Nd UCNPs with different doping amount of Nd^{3+} in $NaGdF_4$:Yb/Nd shell. **e**, Luminescence spectra with different doping amount of

Yb^{3+} in $NaGdF_4$:Yb shell. **f**, Luminescence decay curves of the obtained UCNPs at 808 and 1532 nm under 808 nm excitation. UCNPs were dispersed in cyclohexane for the collection of upconversion emission spectra at an excitation power density of 3.0 W·cm^{-2}. Source data are provided as a Source Data file.

Supplementary Note 6: Energy dissipation process of 808 nm with Nd^{3+} as sensitizer.

Dissipation of 808 nm is related to sensitizers Nd and Er. Since the trichromatic UCNPs used activator Er both in core and middle-part under 808 nm excitation, both of them would emit the same characteristic bands of Er. The emission peaks would be kind of overlapping (**Supplementary Figure 11a**) thus tuning Er concentration in middle part would increase emission of itself, making it hard to quantify the strength of this dissipation process. So that we replaced the emissive center of middle part from Er to Tm to clearly compare the inner emission intensity (**Supplementary Figure 11b**). We found that the existence of emissive center (Tm) has little effect on inner emission intensity (**Supplementary Figure 11c**), it's supposed to be the reason that Tm is not the energy extractor of sensitizer. On steady state, Yb in shell3 and shell4 is the excitation energy extractor that affect equilibrium energy density of sensitizer Nd other than emissive center Tm. We fabricated UCNPs of the same core and dissipation layer thickness of ~ 7 nm as $NaErF_4$@$NaYF_4$@$NaGdF_4$:50%Yb/1%Tm@$NaGdF_4$:10%Yb@$NaGdF_4$:10%Yb/x% Nd (CS_{1-4}, x = 5, 10, 30, 50) UCNPs with different Nd^{3+} doping concentration in dissipation shell S_4 and $NaErF_4$@$NaYF_4$@$NaGdF_4$:50%Yb/1%Tm@$NaGdF_4$:x%Yb@$NaGdF_4$:10%Yb/50% Nd (CS_{1-4}, x = 10, 30, 50, 80) UCNPs with different Yb^{3+} doping concentration in S_3 to investigate the 808 nm excitation dissipation process. Red emission from inner $NaErF_4$ core at ~ 654 nm can be suppressed through increasing Nd^{3+} doping concentration in S_4, but shows little change under 980 and 1532 nm excitations. Increasing Yb^{3+} amount contributes to dissipating more energy through energy transfer from Nd^{3+} to Yb^{3+}. Red emission can also be suppressed but shows little change under 980 or 1532 nm excitations. Luminescence lifetime at 808 nm decreased with increasing sensitizer Nd^{3+} or one of the activators Yb^{3+} but no significant difference at 1532 nm which is from the inner core's emission due to the energy isolation of inert layer $NaYF_4$. This suggest that energy transfer between Nd^{3+} and Yb^{3+} enhanced with increasing doping concentration.

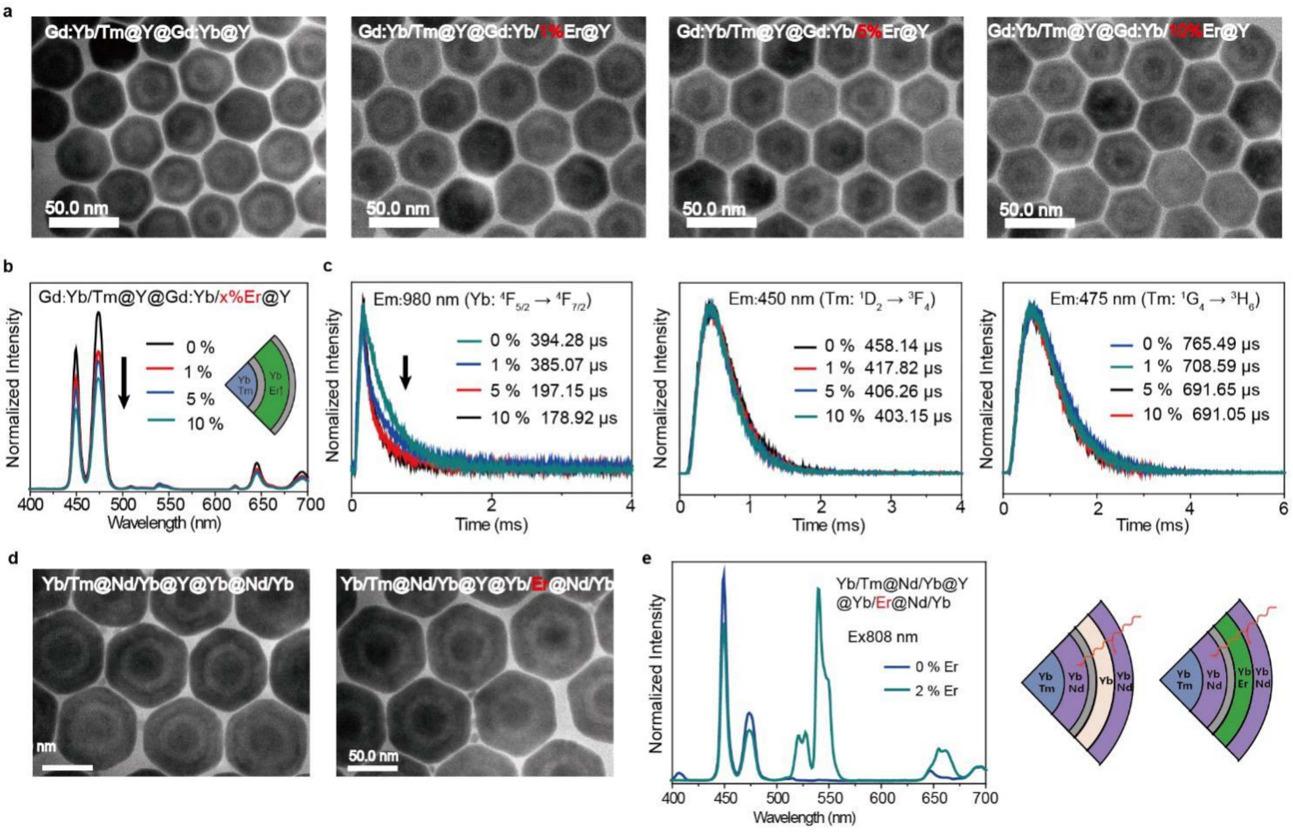

Supplementary Fig. 12. **Energy dissipation process with the same sensitizer in both core and shell and summary of dissipation process. a,** TEM images of $NaGdF_4$:Yb/Tm@$NaYF_4$@$NaGdF_4$:Yb/Er@$NaYF_4$ UCNPs with different Er^{3+} doping amount in $NaGdF_4$:Yb/Er shell (S_2). The synthesis was repeated twice with similar results. **b**, Luminescence spectra at ~ 450/475 nm under excitations of 980 nm of the corresponding UCNPs in **a**. **c**, Luminescence decay curves at 980, 450 and 475 nm of the corresponding UCNPs in **b** under 980 nm excitation. **d**, TEM images and luminescence spectra at ~ 450/475 nm under excitations of 980 nm of $NaGdF_4$:Yb/Tm@$NaGdF_4$:Nd/Yb@$NaYF_4$@$NaGdF_4$:Yb/Er@$NaGdF_4$:Nd/Yb with different Er^{3+} amount in $NaGdF_4$:Yb/Er shell (S_3). The synthesis was repeated twice with similar results. **e**, Table summarized the three cases involved in trichromatic dissipation process. Nanoparticles were dispersed in cyclohexane for collection of upconversion emission spectra at an excitation power density of 3.0 W·cm^{-2} in **b-c** and10.0 W·cm^{-2} in **d**. Source data are provided as a Source Data file.

Supplementary Note 7: Effect of the same sensitizer in both core and shell.

$NaGdF_4$:49%Yb/1%Tm@$NaYF_4$@$NaGdF_4$:20%Yb/x%Er@$NaYF_4$ and $NaGdF_4$:Yb/Er@$NaGdF_4$:Nd/Yb UCNPs with Er^{3+} activators in the dissipation shell were fabricated with same core size and dissipation layers thickness respectively. The 450 and 475 nm blue emission from inner core decreased as introducing more Er^{3+} in dissipation layers indicating the enhanced

dissipation efficiency. Luminescence lifetime at 980 nm also decreased as increasing Er^{3+} doping concentration in dissipation shell but no significant lifetime difference was observed for the emissions at ~ 450 and ~ 475 nm from inner core.

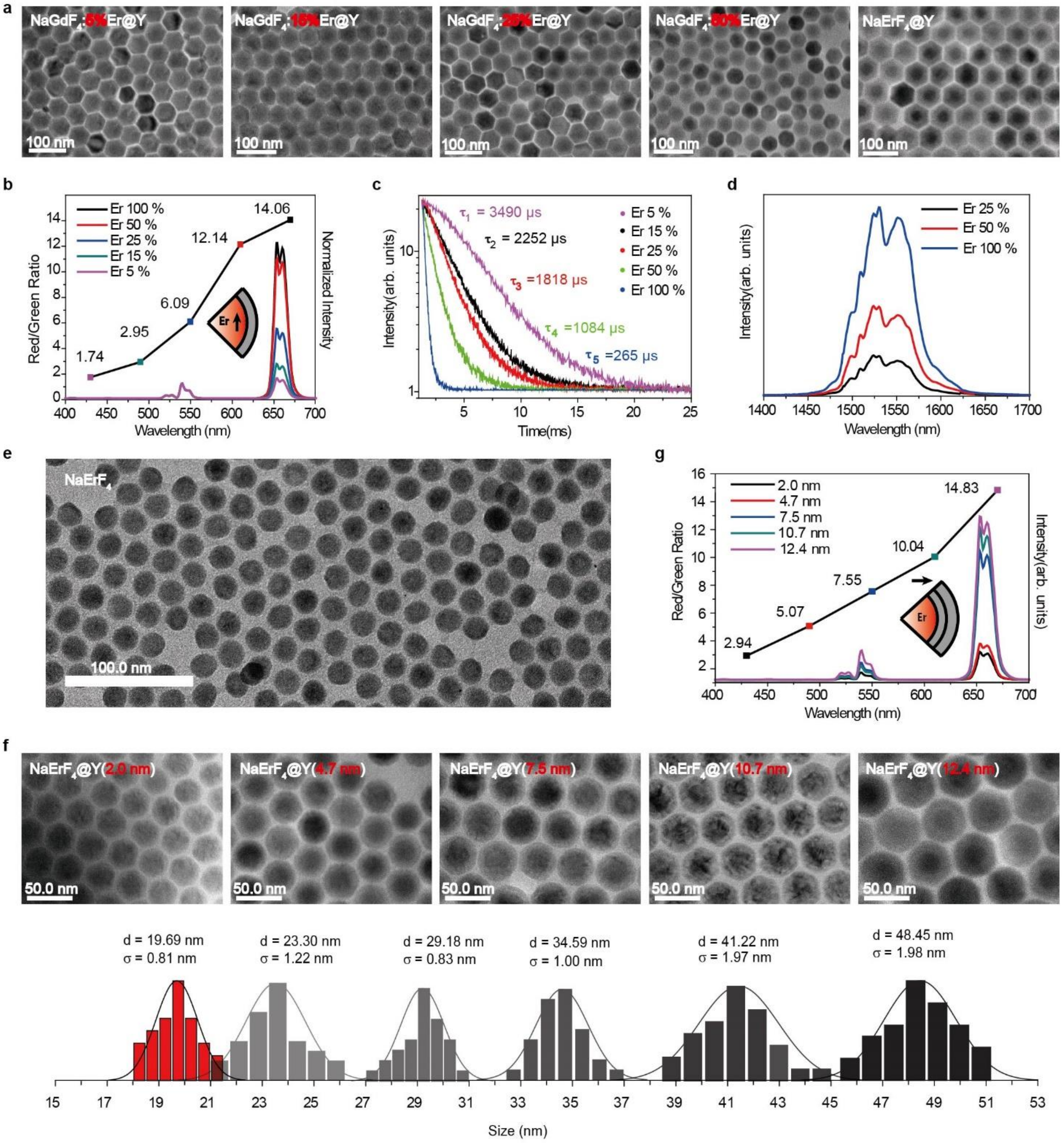

Supplementary Fig. 13. **NaErF4 core for pure red emission.** **a**, TEM images of $NaGdF_4$:x%Er@$NaYF_4$ UCNPs with different Er^{3+} doping concentration in the core. All synthesis related to the core red emission were perform twice with similar result. **b**, Luminescence spectra of the $NaGdF_4$:x%Er@$NaYF_4$ UCNPs with corresponding red/green ratio plot under 1532 nm excitation. **c**, Luminescence decay curves at ~ 654 nm under 1532 nm excitation. **d**, Luminescence spectra of $NaGdF_4$:Er x%@$NaYF_4$ nanoparticles at 1532 nm under the excitation of 980 nm. **e-f**, The distribution histograms with mean diameter and standard deviation were obtained from the statistical results of more than 30 particles. **g**, Luminescence spectra with corresponding red/green ratio plot of the obtained UCNPs under 1532 nm excitation. All the nanoparticles were dispersed in cyclohexane for the

collection of emission spectra at an excitation power density of 5.0 W·cm^{-2}. Source data are provided as a Source Data file.

Supplementary Note 8:Coupling cross-relaxation and surface passivation for pure red emission.

In the $NaGdF_4$:x%Er@$NaYF_4$ (C_{1-2}, x= 5, 15, 25, 50, 100) UCNPs with same core size and shell thickness, it is found that red emission band at ~ 654 nm was enhanced and red/green ratio increased 8 folds with increasing of Er^{3+} doping concentration under excitation of 1532 nm. The high doping concentration of Er^{3+} ions possess dual functions for harvesting excitation energy of 1532 nm and performing red emission at ~ 654 nm. Due to the enhancement of cross-relaxations among Er^{3+}, luminescence lifetime at 654 nm decreased along with increasing of ~1532 nm emission under the excitation of 980 nm. Thickness of $NaYF_4$ inert shell also plays an important role for 654 nm dominated red upconversion emission. The transition $^4F_{9/2}$→$^4I_{15/2}$ related to red emission is much more sensitive to surface defects than $^4S_{3/2}$→$^4I_{15/2}$ transition related to green emission under 1532 nm excitation, which means a thicker passivation shell contribute to a pure red emission. For a pure red emission, $NaErF_4$ core with a thick $NaYF_4$ passivation shell of ~ 10 nm was chosen to construct the trichromatic UCNPs.

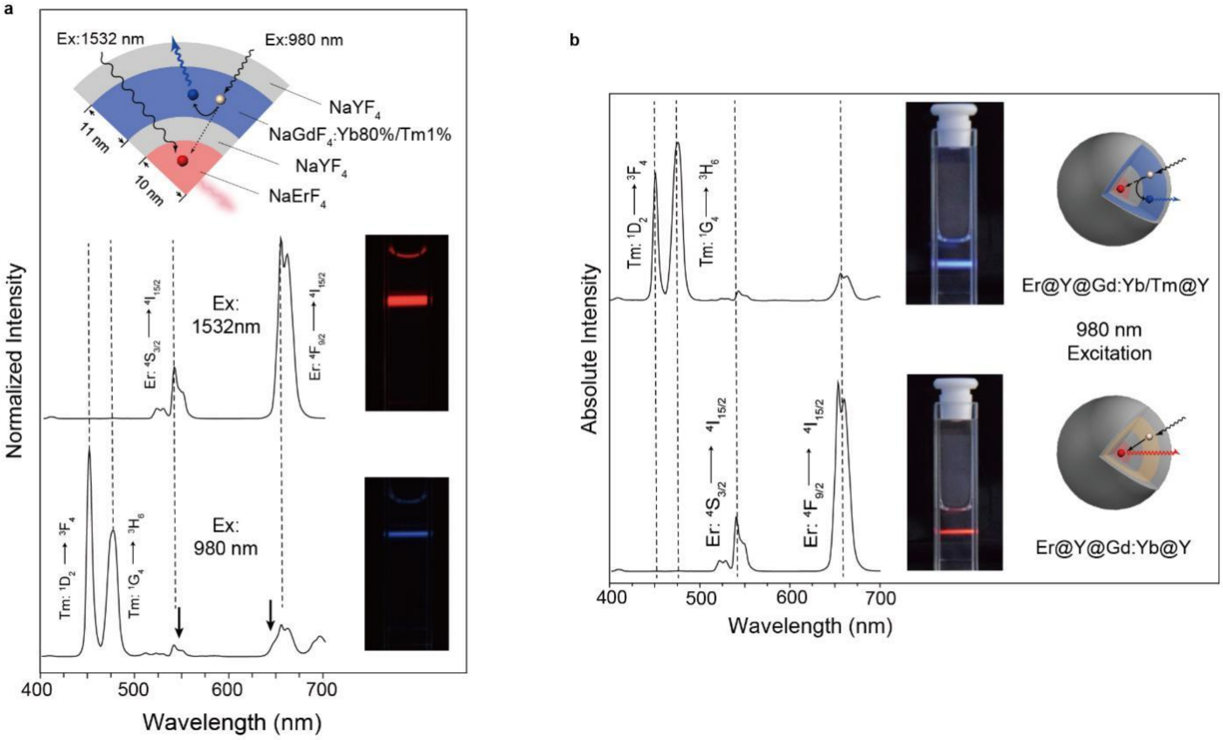

Supplementary Fig. 14. Blue and red dual-emission UCNPs. a. Emission spectra of UCNPs with optimized structure under 980 nm excitation and 1532 nm excitation. **b**, Emission spectra of UCNPs with or without activator doping dissipation shell as a comparison under 980 nm excitation. UCNPs of nanostructure $NaErF_4$@$NaYF_4$@$NaGdF_4$:80%Yb/1%Tm@$NaYF_4$ with core radius ~10 nm and shell thickness ~11 nm are constructed as optimized structure. This structure realized blue and red dual-responsive emission. As a comparison, UCNPs nanostructure $NaErF_4$@$NaYF_4$@$NaGdF_4$:80%Yb@$NaYF_4$ are constructed and performs intense red emission from the inner core under the same 980 nm excitation power density. All the nanoparticles were dispersed in cyclohexane for the collection of emission spectra under the excitations 1532 nm (5.0 W·cm^{-2}) and 980 nm (1.5 W·cm^{-2}) respectively.

Supplementary Note 9:Interpretation of the energy dissipation mechanism.

The energy dissipation process is proposed to dominate the filtration effect. Thus, manipulating incident photon flux in typical core-shell structure includes three main factors. (1) The ground state absorption (GSA) of sensitizers (Yb^{3+}, Nd^{3+}, etc.), which should have large absorption cross-section at corresponding wavelength, inhibits incident excitation energy into the inner core. This can be enhanced by increasing sensitizer doping amount. (2) According to energy transfer (ET) effect, activators (Tm^{3+}, Er^{3+}, etc.) doping favors energy extraction from sensitizers, which is indicated by the decrease of sensitizer lifetime. Increasing activator doping concentration enhances the energy transfer process and cross-relaxation among dopants thus further consumes excitation energy. (3) Thickness of dissipation layer is key to mediate energy dissipation efficiency as well. Excitation energy depletes gradually with an increasing thickness of energy dissipation layer with sensitizers and activators.

Supplementary Note 10: Interpretation of the change of sensitizer lifetime and de-saturation of sensitizer.

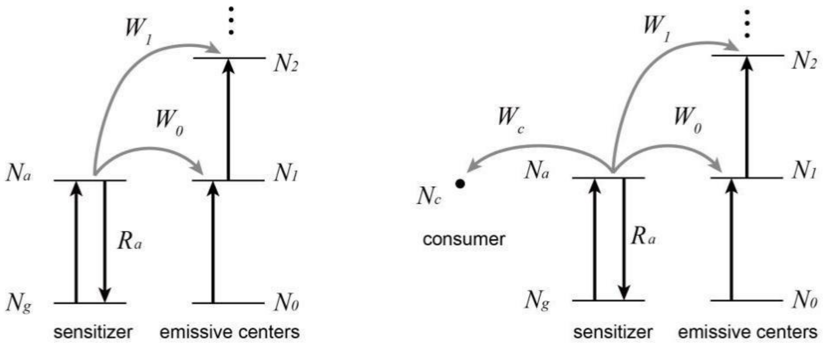

For a simplified multiple-step energy transfer upconversion process, the time-resolved rate equation of energy states of sensitizers Yb^{3+} can be derived as follows:

$$-\frac{dN_g}{dt} = \frac{dN_a}{dt} = \frac{\sigma P}{h\nu} N_g - \tau^{-1} N_a$$

Where N_g and N_a present the population density of ground state and excited state of Yb^{3+}. τ is the decay time of excited state, σ is the absorption cross-section of sensitizers, P stands for excitation power density, h is Planck's constant and ν is the frequency of excitation light.

At steady state with a constant total density N of Yb^{3+} ions

$$-\frac{dN_g}{dt} = \frac{dN_a}{dt} = 0$$

$$N_a = N - N_g$$

And the decay time of Yb^{3+} is

$$\tau = \frac{1}{\sum_i W_i N_i + R_a}$$

Where N_i is the active state population of activators and W_i stands for energy transfer rate from

sensitizer to activators' correspondent transition. R_a is the emission rate constants of excited state. Thus, the relationship of Yb^{3+} excited state population density N_a and excitation power density P can be derived as:

$$N_a = \frac{\frac{\sigma P}{hv} N}{\sum_i W_i N_i + R_a + \frac{\sigma P}{hv}} = \frac{P}{A + B \cdot P}$$

Where A and B is simplified from $(\sum_i W_i N_i + R_a)\frac{hv}{\sigma N}$ and $\frac{1}{N}$. With a constant total density of Yb^{3+} ions, B is definite. Introducing more consumers in the energy transfer system, decay rate of Yb^{3+} excited state is increased to $\sum_i W_i N_i + R_a + W_c N_c$ thus A is increased to $(\sum_i W_i N_i + R_a + W_c N_c)\frac{hv}{\sigma N}$. The decay time of Yb^{3+} is thus reduced to $\tau = \frac{1}{\sum_i W_i N_i + R_a + W_c N_c}$. This result is consistent with our experiment of 980 nm emission lifetime in main text.

In the excitation of core-shell UCNPs, the unconverted photons will penetrate the outer layers and get into the inner core. The emission of Er^{3+} is positively correlative with the incident photons. The rate of photons that entering the core is:

$$w = w_0 - w_1$$

where w_0 is the rate of incident photons and w_1 is the rate of converted photons in outer layer. The energy absorbing rate is σP , where σ is the absorption cross-section and P is the excitation power density. But only sensitizers in ground state of outer layer are able to convert the photons, so that the photon converting rate of sensitizer in outer layer is:

$$w_1 = \frac{\sigma P}{hv} N_g = \frac{\sigma P}{hv}(N - N_a)$$

It means that lowering the outer layer sensitizer activated population N_a can increase the converting rate w_1 under the same excitation power and subsequently reducing the photons approaching the inner core (w).

Tm^{3+}, as the energy consumer, is able to extract energy from Yb^{3+} in the activated state allowing Yb^{3+} returns to ground state. When increasing the excitation power P, the activated state population N_a will increase to the total population N. Doping of Tm delays this saturation process by adding extra energy transfer routes. Consequently, at a specified excitation power density P, the population density of active state N_a is decreased with the increasing coefficient A thus reducing the rate of photons approaching to inner core w.

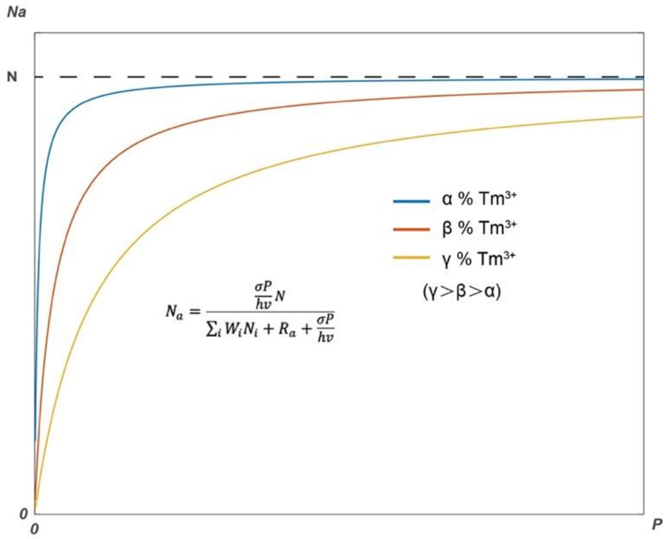

Simulation curves of sensitizer activated state population as a function of excitation power density with different Tm doping concentration.

These results indicate that excitation energy distribution can be tuned with altering the component of dissipation shell. A specific amount of sensitizers with more activators results to the reduction of active state population density favoring the energy trapping process. More excitation energy transfers from sensitizers to emissive centers leads to higher dissipation efficiency. As a result, excitation photons reach to inner core is reduced thus suppressed the inner core’s emission.

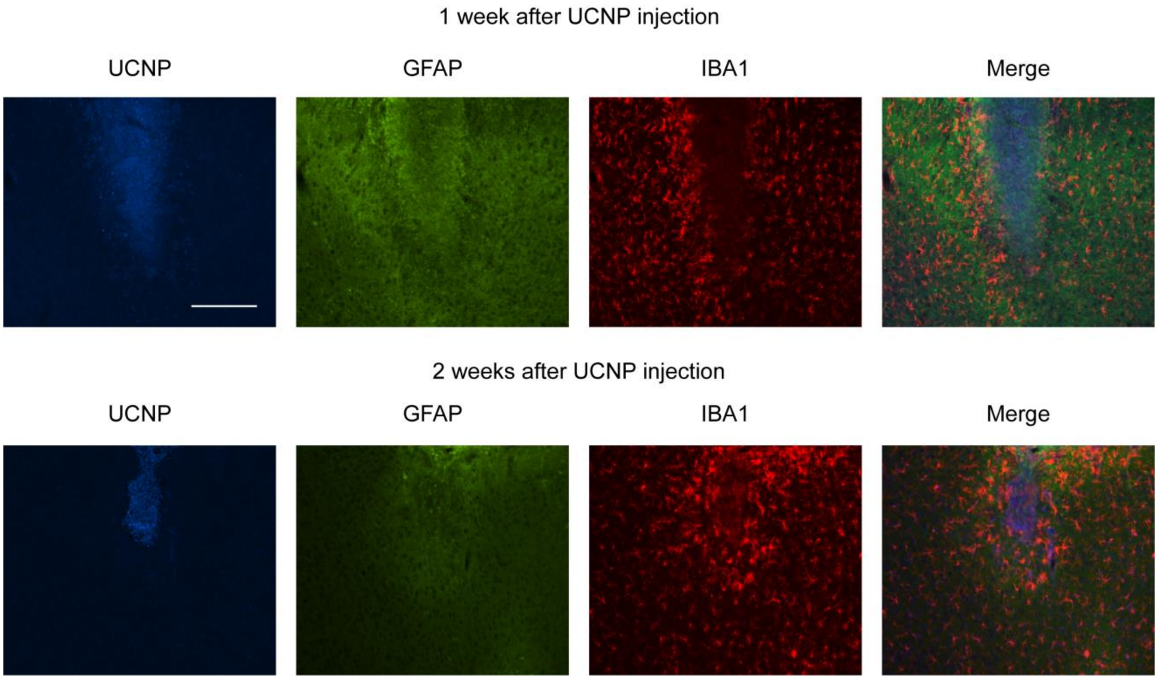

Supplementary Fig. 15. **Immunochemistry staining results of GFAP and IBA1 after UCNPs injection.** Upper panel, GFAP and IBA1 immunostaining of the injection site from mouse after one week of UCNPs injection. Lower panel, GFAP and IBA1 immunostaining of the injection site from mouse after two weeks of UCNPs injection. UCNPs were observed under 408 nm excitation laser. Scale bar, 200 μm. Immunohistochemistry was repeated for 2 times and similar results were observed.

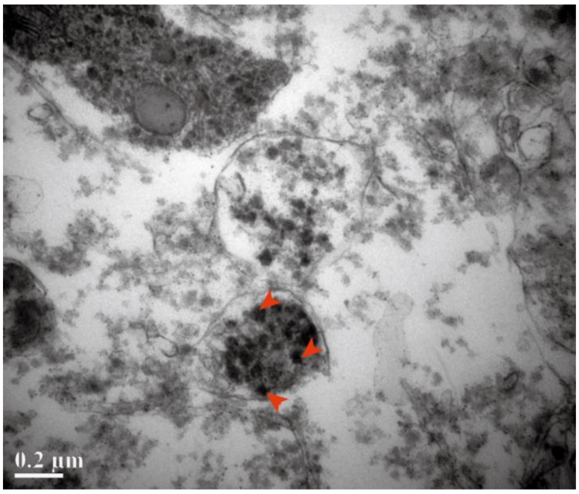

Supplementary Fig. 16. Electron micrographs of UCNPs in M2. TEM photos of UCNPs in M2. Red arrows indicate UCNPs. One sample tissue was processed.

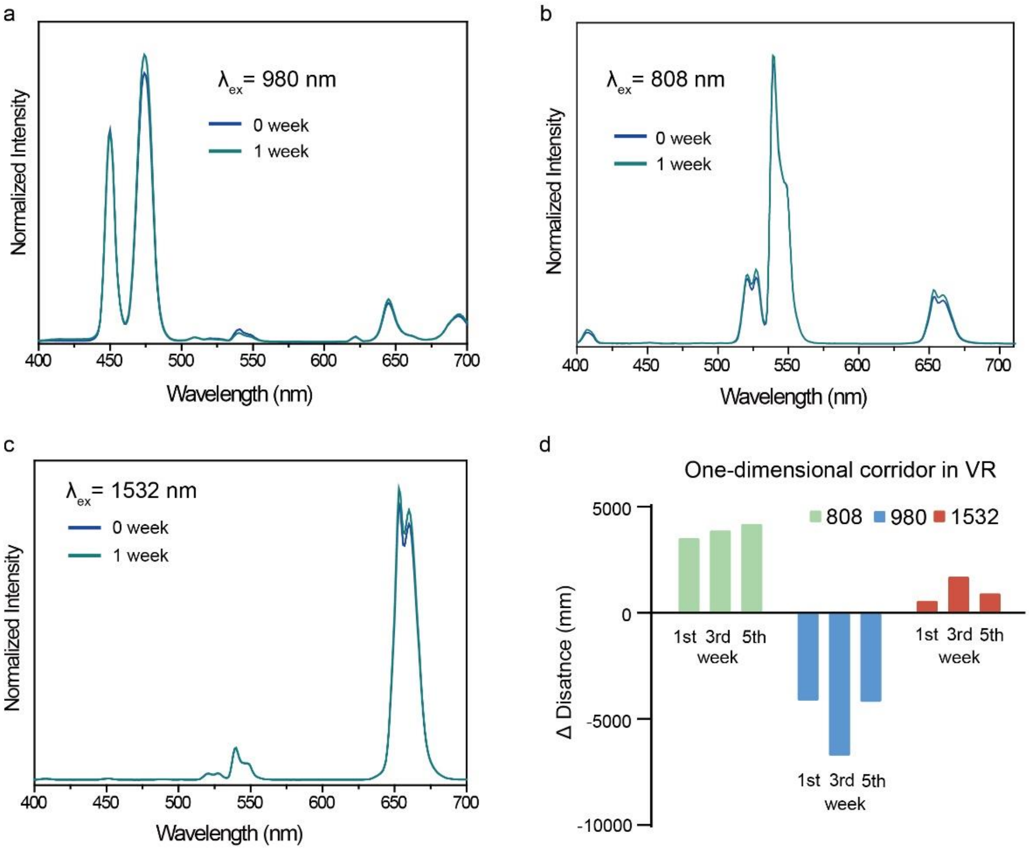

Supplementary Figure 17. Physiological stability of UCNPs. a-c, Emission spectrum of UCNPs under 808 nm, 980 nm and 1532 nm excitation before and after stocked in ACSF for a week. **d**, Exemplar behavioral performances of mice at 1st, 3rd and 5th week after UCNP injection. Each column represents for a single trial of one mouse. And only one trial of each mouse was recorded one day. Source data are provided as a Source Data file.

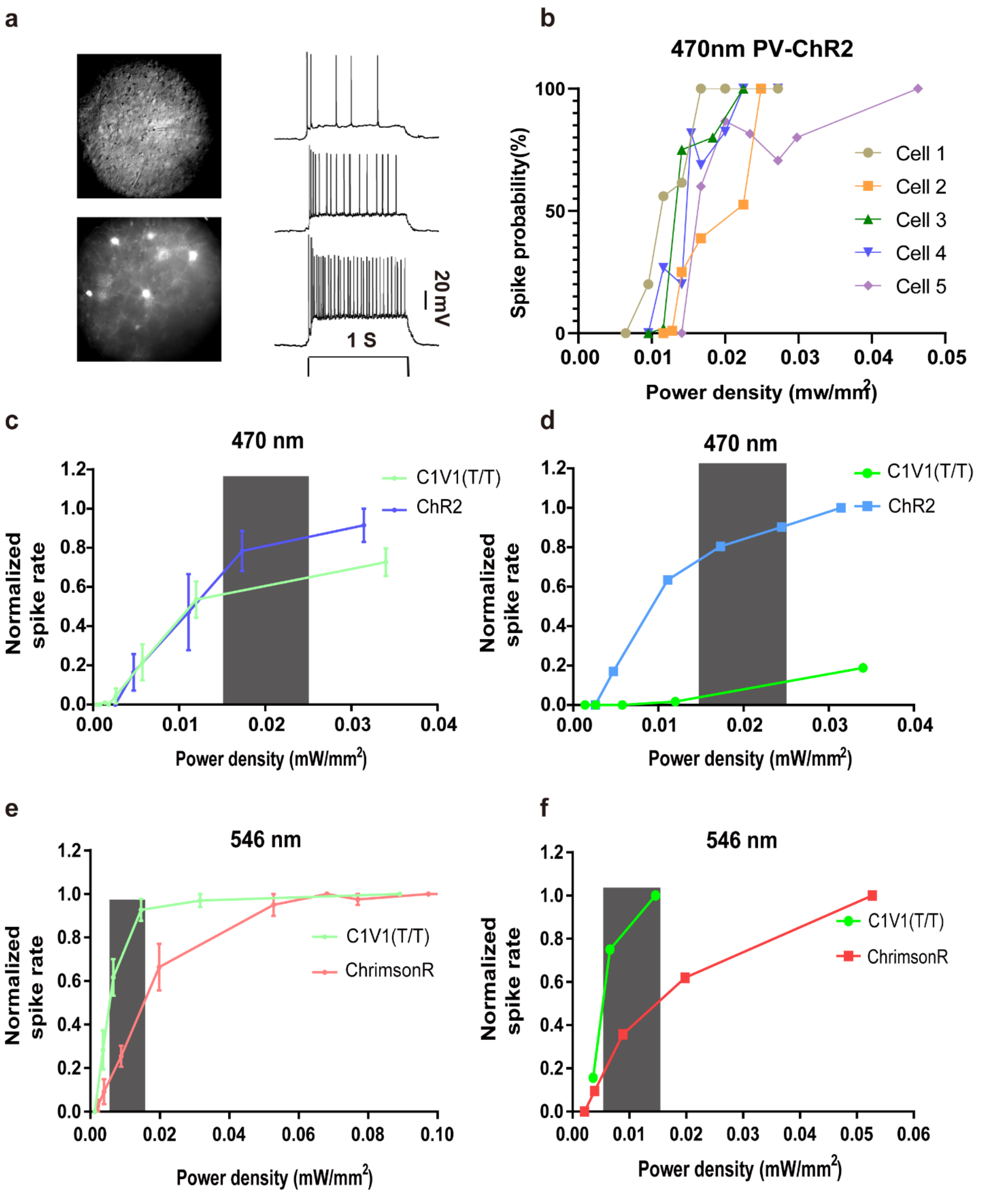

Supplementary Fig. 18. Visible light excitation of neural populations in mouse M2 brain slice. a, Left, representative of phase contrast (top) and mCherry (bottom) fluorescent images of ChrimsonR-mCherry fusion transfected neuron. Scale bar, 100 µm. Right, responses of ChrimsonR-expressing cell to 665 nm emission. (top: 0.017958 mW/mm^2, middle: 0.033004 mW/mm^2, bottom: 0.07411 mW/mm^2). Fluorescent image was taken every time before patching the neuron. **b,** Example responses of 470 nm activation of ChR2-expressing cell with a 5 ms light pulse duration. **c,** Normalized responses

of C1V1-expressing cells (n = 6) and ChR2-expressing cells to 470 nm emission (n = 3) with 1 s duration. Data are presented as mean values ± SEM. **d,** Example of ChR2-(C1V1-) expressing cell to 470 nm emission with 1 s duration. **e,** Normalized responses of C1V1-expressing cells (n = 9) and ChrimsonR-expressing cells to 546 nm emission (n = 5) with 1 s duration. Data are presented as mean values ± SEM. **f,** Example of C1V1-(ChrimsonR-)expressing cell to 546 nm emission with 1 s duration. Source data are provided as a Source Data file.

a

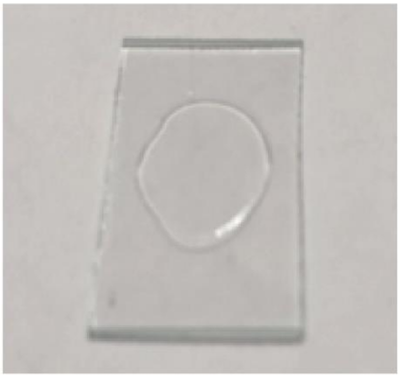

b

Thickness of ACSF (mm)	Average Detected Power (mw)		
	808-nm	980-nm	1532-nm
0	511.0	481.0	527.2
2	518.1	450.2	4.8
3	519.2	439.3	6.7

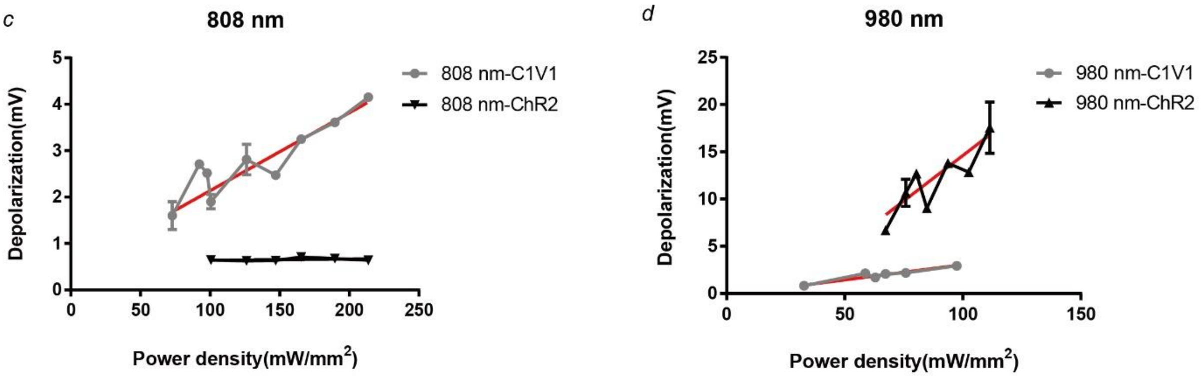

Supplementary Fig. 19. Subthreshold responses of ChR2- and C1V1-expressing cells to NIR lasers. a, Image of transparent solid UCNPs film. **b**, Powers of NIR lasers transmitted through ACSF of different thickness. **c**, Subthreshold responses of ChR2- and C1V1-expressing neurons to 980 nm emission (n = 3 independent neurons from mice) with 1 s duration. Data are presented as mean values ± SEM. **d**, Subthreshold responses of ChR2- and C1V1-expressing neurons to 808 nm emission (n = 3 independent neurons from mice) with 1 s duration. Data are presented as mean values ± SEM. Source data are provided as a Source Data file.UCNPs diffused in the solution of recording chamber, so that the concentration could not be maintained in patch clamp experiment. The transparent solid PMMA film was used to fixed the UCNPs (**Supplementary Fig.19a**). Due to the strong absorption of water to 1532 nm light, brain slice experiments under 1532 nm excitation couldn't be conducted.

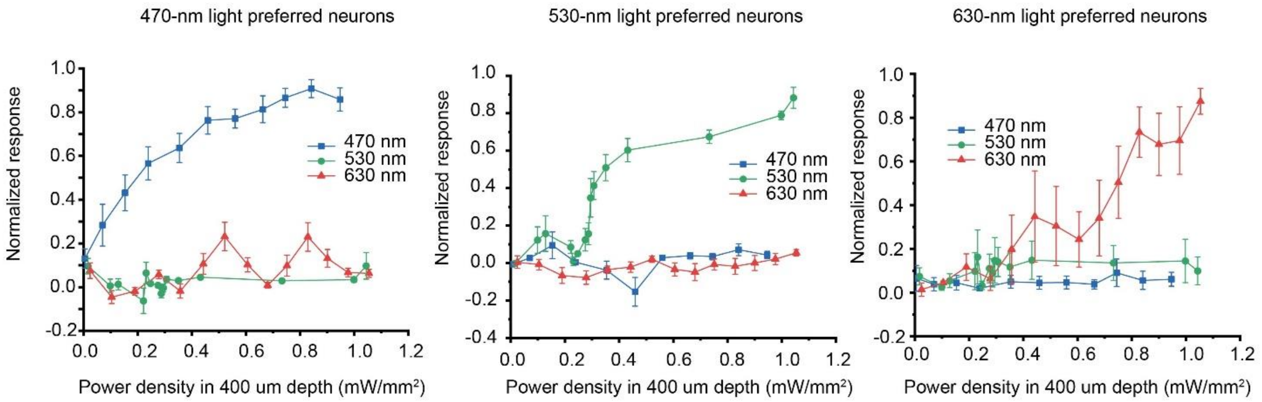

Supplementary Fig. 20. **Optogenetic protein chromatic selectivity under different power density of red, green and blue visible light lasers in mouse V1 neurons *in vivo*.** Normalized responses of neurons with 470, 530 and 630 nm laser under different power intensities. Left, 470 nm light preferred neurons (*n* = 8 neurons). Middle, 530 nm light preferred neurons (*n* = 10 neurons). Right, 630 nm light preferred neurons (*n* = 4 neurons). Data are presented as mean values +/- SEM

We recorded neuron responses with 470, 530 and 630 nm lasers under different intensities, and divided them into three populations according to their preferred responses to specific light.

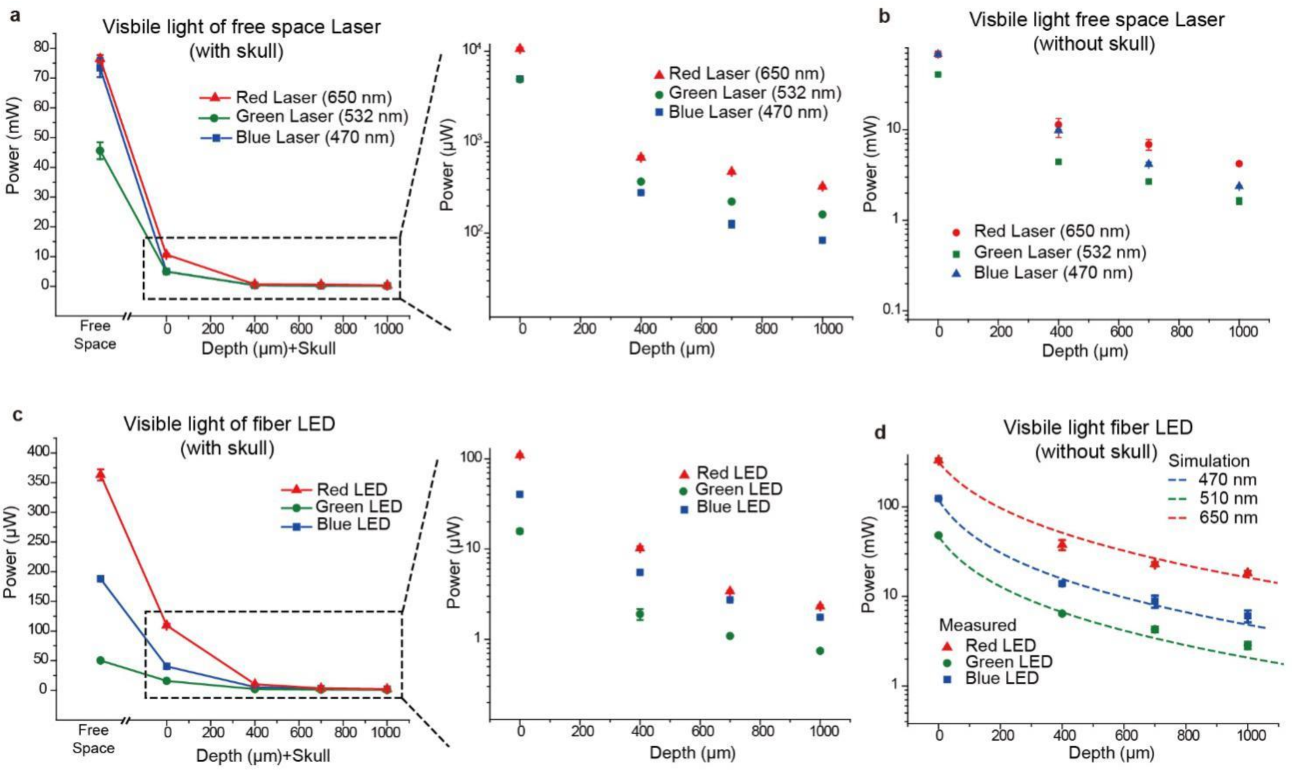

Supplementary Fig. 21. **Visible lights and NIR lights penetration test of brain tissue. a-b**, Measurement of laser power intensity in brain tissue with or without skull at different depth. n =3 independent brain slice samples at each depth. **c-d**, Measurement and simulation of fiber LED power intensity in brain tissue with or without skull at different depth. n =3 independent brain slice samples at each depth. Data are presented as mean values +/- SEM The excitation powers are the same to electrophysiological experiments as in Fig. 4 of main text. Source data are provided as a Source Data file.

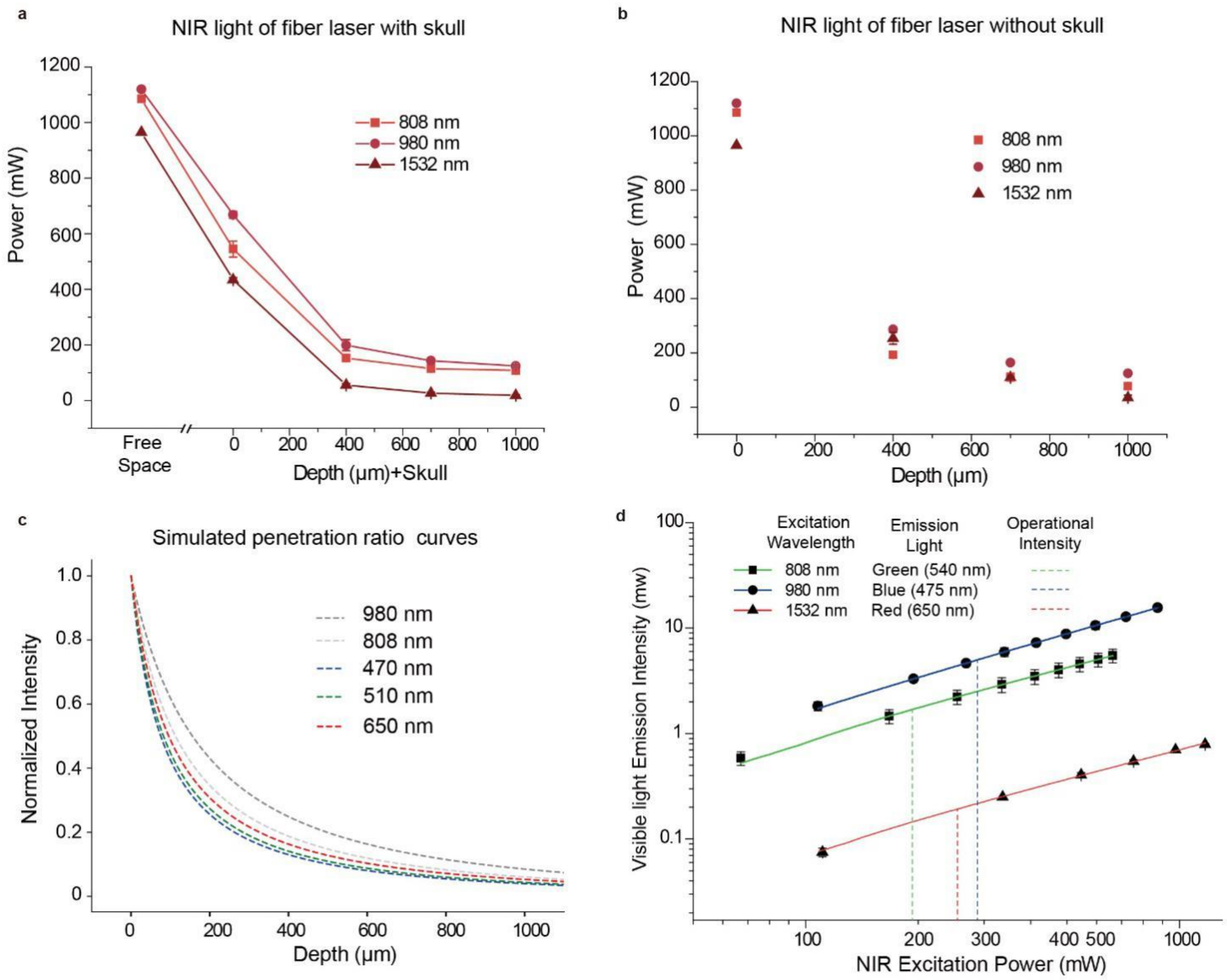

Supplementary Fig. 22. NIR lights penetration test of brain tissue and upconversion efficiency. a-b, Measurement of NIR light power intensity in brain tissue with or without skull at different depth. n=3 independent brain slice samples at each depth. Data are presented as mean values +/- SEM**. c,** Simulation of normalized intensity ratio of light at 980 nm, 808 nm ,650 nm, 510 nm and 470 nm penetrating brain tissue of different depth without skull. The scattering simulation of 1532 nm is not suitable for the absorption dominated model because of the intense water absorption[3].(Details in Methods.) **d,** Upconversion emission power of trichromatic UCNPs (0.75 mg, 750 mg/mL in 1 L saline) as a function of corresponding NIR laser power. n = 3 independent UCNPs test samples. Data are presented as mean values +/- SEM The power conversion efficiencies for 980 nm to blue emission, 808 nm to green emission and 1532 nm to red emission are 1.77%, 0.84% and 0.07% respectively. The dash line indicates the NIR power used in the experiments. Source data are provided as a Source Data file.

Supplementary Note 11:NIR penetration test and upconversion efficiency .

The light intensity used in chromatic selectivity experiments were 0.44 mW/mm^2 at 470 nm, 0.07

mW/mm^2 at 530 nm and 0.83 mW/mm^2 at 650 nm at the brain surface. To prevent the chromatic cross-talk of different optogenetic proteins in ChrimsonR- and C1V1-expressing neurons, we measured the upconversion efficiency and kept the intensity of the 980, 808 and 1532 nm NIR excitations below 89.1 mW/mm^2, 86.4 mW/mm^2 and 76.8 mW/mm^2 at the brain surface (Methods). Note that the visible light intensity used is limited to the LED power, the permissible intensity of visible light for chromatic selectivity could be higher.

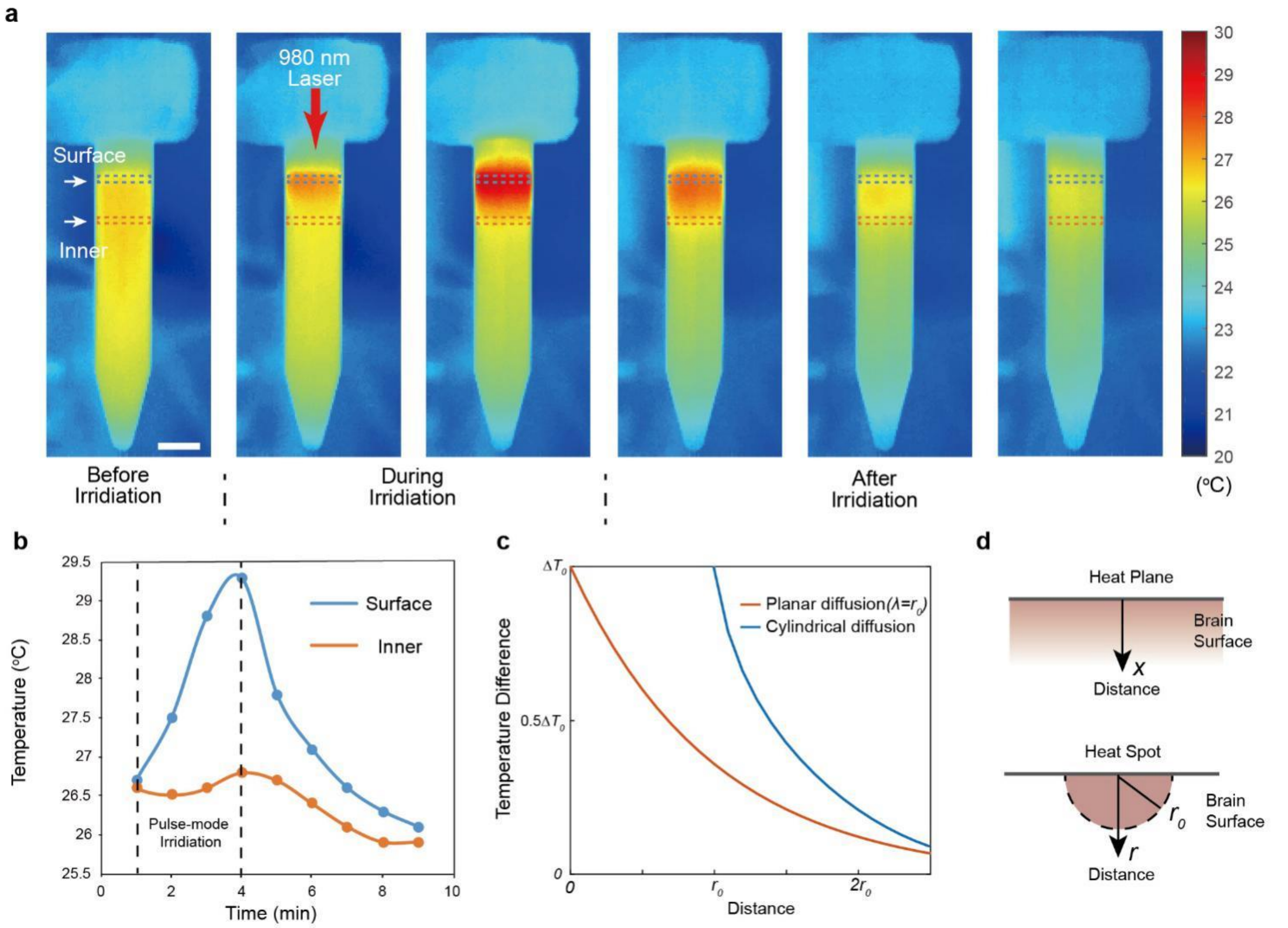

Supplementary Fig. 23. **Temperature comparison of tissue at surface and inner part. a**, Thermal image photographs of temperature distribution of 1% intralipid before, during and after irradiation. Scale bar = 1 cm. Sample was first heat to 26 ºC with uniform distribution. **b,** Temperature at surface and inner part as a function of time. Results are calculated from the average value in the corresponding area (Rectangle marked with dot lined) in **a**. Source data are provided as a Source Data file. **c**, Simulation of temperature difference with planar heat diffusion or cylindrical heat diffusion as a function of distance from the brain surface. **d**, Illustration of planar and cylindrical heat diffusion in brain tissue.

Supplementary Note 12: NIR thermal effect induced heat distribution between brain surface and local tissue.

The brain surface temperature is much higher than inner local temperature with a fluctuation during light exposure (**Supplementary Figure 23a,b**). So that we use temperature recorded from the brain surface as representative for the highest brain tissue temperature increase. For standard one-dimensional model, the heat source surface is regarded as a surface directly contact with the brain (**Supplementary Figure 23d**). In such arrangement, temperature as a function of time t and distance x from the surface can be described with an additional term of heat flow induced by blood perfusion[5]

$$c_B \frac{\partial \Delta T}{\partial t} = k_B \frac{\partial^2 \Delta T}{\partial x^2} - \omega c \Delta T$$

where c_B and k_B are the specific heat capacity and thermal conductivity of the brain respectively. ω and c are blood flow rate and perivascular tissue thermal conductivity respectively. ΔT is the temperature difference compared with normal tissue, which is that we're interested in. In steady state, this equation gives an exponential decay solution of distance (**Supplementary Figure 23c orange line**):

$$\Delta T(x) = \Delta T_0 e^{-\frac{x}{\sqrt{\frac{k_B}{\omega c}}}} = \Delta T_0 e^{-\frac{x}{\lambda}}$$

where ΔT_0 is the temperature difference at contact surface and the constant λ roughly indicates the average diffusion length of temperature induced by heat.

When considering cylindrical thermal diffusion, the heat spot can be regard as a point source with radius of r_0 (**Supplementary Figure 23c blue line**). The function given in cylindrical coordinates follows the below steady state equation[6]:

$$\frac{1}{r}\frac{d}{dr}\left(r\frac{d\Delta T}{dr}\right) - \lambda^2 \Delta T = 0$$

where λ is as the same as length constant in planar diffusion, r is the radial distance from the heat source center. The boundary condition is $\Delta T(r_0) = \Delta T_0, \Delta T(\infty) = 0$ .The solution of this equation is:

$$\Delta T(r) = \Delta T_0 \frac{K_0(\frac{r}{\lambda})}{K_0(\frac{r_0}{\lambda})}$$

In which, K_0 is the zero order Bessel function of the second kind. Both of models show an exponentially decline of temperature difference as a function of distance from the brain surface. It suggests that limiting the temperature change at brain surface can make sure the local region will not be overheating.

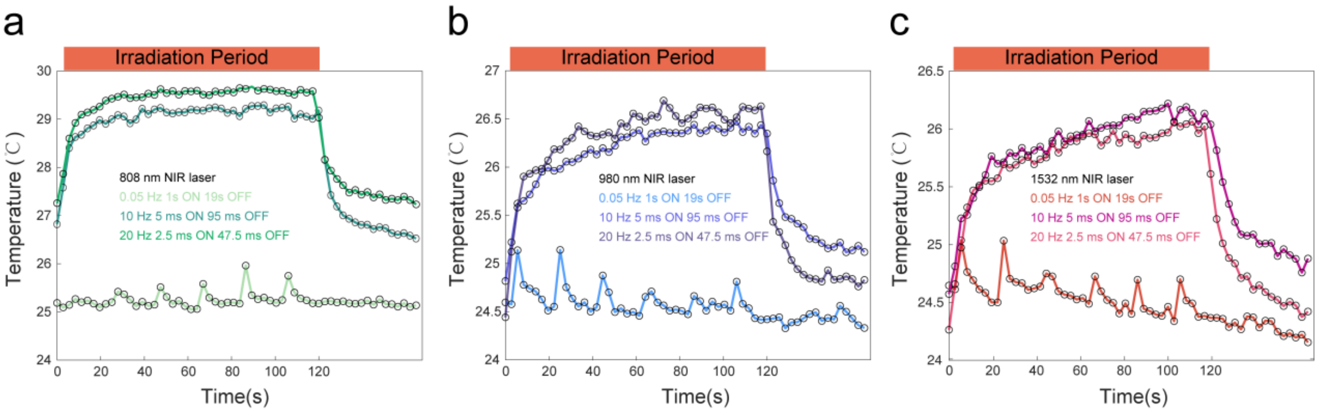

Supplementary Fig. 24. Curves of temperature rise of stimulation of different frequencies. a, Curves of temperature rises of stimulation of 808 nm laser of 0.05 Hz, 10 Hz and 20 Hz (120 s in total). **b,** Curves of temperature rises of stimulation of 980 nm laser of 0.05 Hz, 10 Hz and 20 Hz (120 s in total). **c,** Curve of temperature rises of stimulation of 1532 nm laser of 0.05 Hz, 10 Hz and 20 Hz (120 s in total). Source data are provided as a Source Data file.

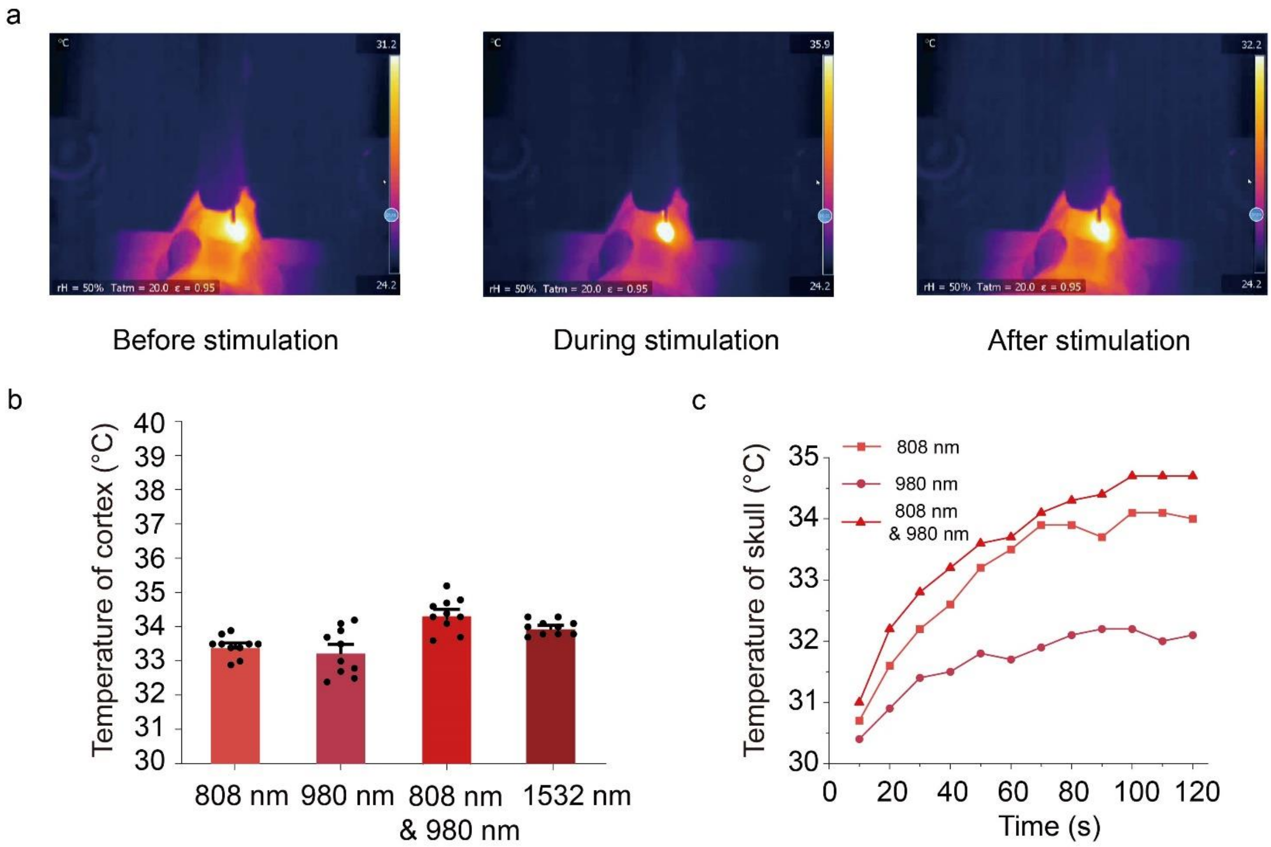

Supplementary Fig. 25. **Temperature increase under NIR light excitation on brain surface. a,** Infrared thermal imaging results of fixed mouse before, during and after NIR light stimulation. **b,** Temperature of cortex surface without skull under 808 nm, 980 nm, 808nm & 980 nm and 1532 nm NIR excitation (conditions for *in vivo* electrophysiology, pulse duration = 1 s, interval = 19 s, Power densities of NIR laser were 86.40 mW/mm^2 for 808 nm, 89.10 mW/mm^2 for 980 nm and 76.8 mW/mm^2 for 1532 nm). The temperature test is repeat for 10 times. **c,** Temperature increase of skull surface under 808 nm, 980 nm or 808nm & 980 nm NIR excitation (conditions in behaviour experiments, pulse duration = 0.1 s, interval = 0.1 s, Power densities of NIR laser were 59.10 mW/mm^2 for 808 nm, 38.90 mW/mm^2 for 980 nm, temperature was detected at every 10 s, total duration = 120 s).

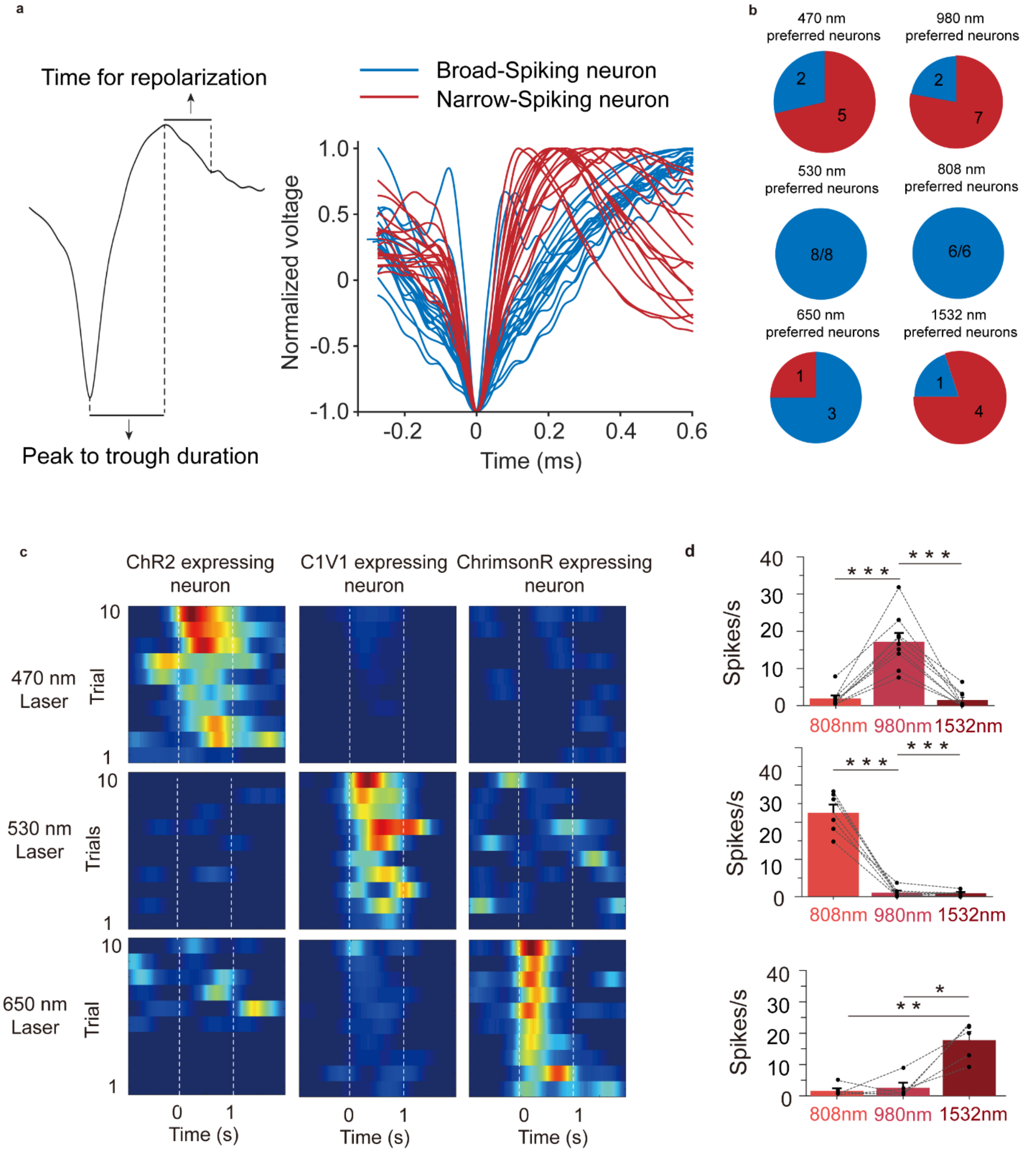

Supplementary Fig. 26. **Spike waveforms of all recorded neurons. a**, The schematic for analyzing waveform and templates of all recorded neurons which were divided into Broad-Spiking and Narrow-Spiking neurons. **b**, Proportion of two groups neurons in all recorded neurons according to the waveforms. **c**, Representative optogenetic responses in ChR2-expressing (left), C1V1-expressing (middle) and ChrimsonR-expressing neurons stimulated by visible lasers at 470, 530 and 630 nm, respectively (0.06 mW/mm^2 for 470 nm, 0.008 mW/mm^2 for 530 nm and 0.14 mW/mm^2 for 630 nm at 400 μm depth). **d**, Average responses of neurons activated by NIR light at 808 nm, 980 nm and 1532 nm (n_{808nm} = 6 neurons in 5 mice, n_{980nm} = 9 neurons in 6 mice, n_{1532nm} = 5 neurons in 4 mice, **P <

0.01; ***P < 0.001, one-way repeated measures Analysis of variance (ANOVA) with Tukey's multiple comparison test). The exact p values are listed in Table S3. Data were presented as mean ± s.e.m. Source data are provided as a Source Data file.

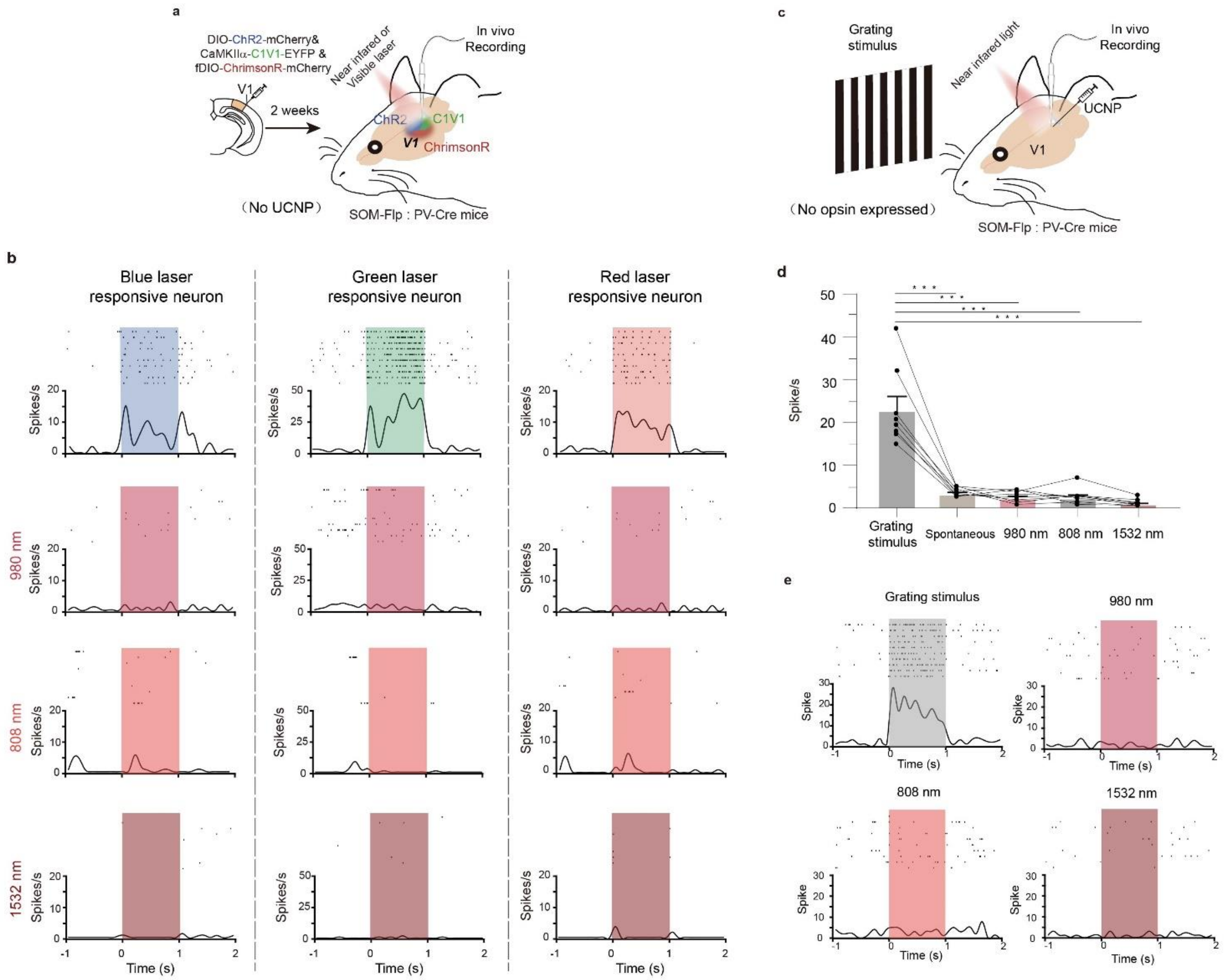

Supplementary Fig. 27. **Electrophysiological contrast tests without UCNPs or opsins expressing.** **a**. Schematics of experiment design with opsin expressing but without UCNPs. **b**. Example raster plots of blue laser responsive (left), green laser responsive (middle) and red laser responsive (right) neurons under 980 nm,808 nm and 1532 nm NIR laser in **a**. **c**. Schematics of experiment design with UCNPs injected but without opsins expressing in distinct neurons. **d**. Average firing rate of recorded neurons with grating stimulus, spontaneous, 980 nm, 808 nm and 1532 nm NIR light in **c** (n = 8 neurons in 2 mice, ***P<0.001, two side paired t-test). Data are presented as mean values ± SEM. **e**. Example raster plot of recorded neuron with grating stimulus, 980 nm, 808 nm and 1532 nm NIR light. Source data are provided as a Source Data file.

a

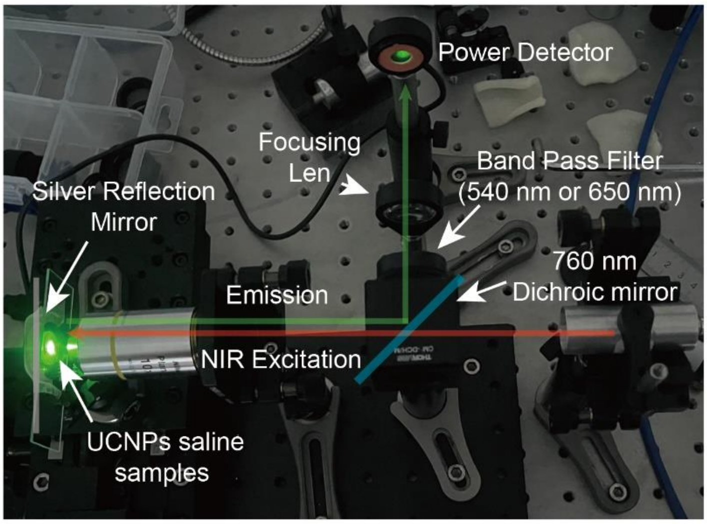

b

Wavelength	540nm	650nm	R/G Ratio
Power (μW)	17.59	5.94	0.337
	43.2	12.38	0.287
	59.6	18.5	0.310
	76.3	25.5	0.334

c

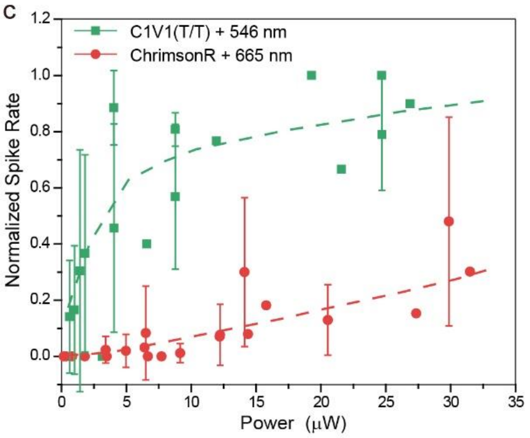

Supplementary Fig. 28. Comparison of green/red emission ratio under 808 nm excitation with C1V1(T/T) and ChrimsonR response ratio. a, Photos of UCNPs emission power measurement set up. **b**, Table of measured visible light power under the same laser output to in vivo experiments. **c**, Recorded cellular level neuronal activities by patch clamp from brain slices expressed C1V1 and ChrimsonR respectively under different visible light powers (n = 9 for C1V1 expressing-cells and n = 5 for ChrimonsR expressing-cells). Data are presented as mean values ± SEM.

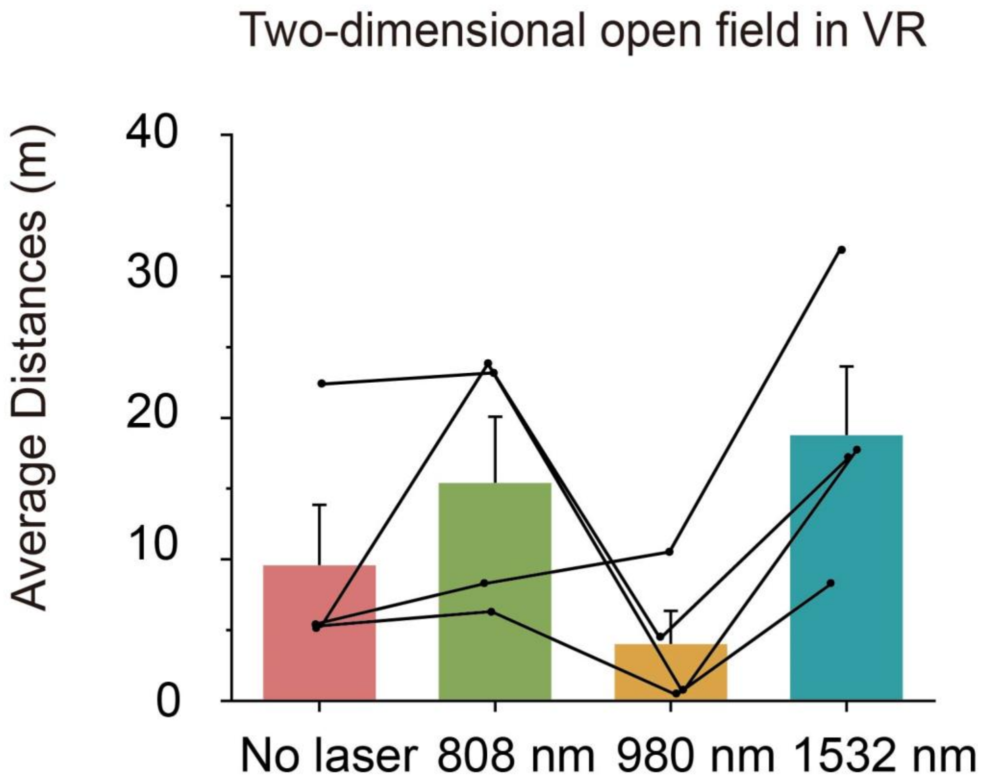

Supplementary Fig. 29. Test of three-color optogenetic manipulation in open field tasks. Mice in Fig. 4b were used in this experiment. Average running distances of locomotion movement in two-dimensional open field in VR system were measured under 808 nm, 980 nm and 1532 nm NIR excitations (*n* = 4 mice; one-way repeated measures ANOVA with Tukey's multiple comparison test; 59.1 mW/mm^2 for 808 nm, 38.9 mW/mm^2 for 980 nm, 76.8 mW/mm^2 for 1532 nm at brain surface, 100 ms pulse width, 5 Hz, total duration = 120 s). Data are presented as mean values ± SEM. Source data are provided as a Source Data file.

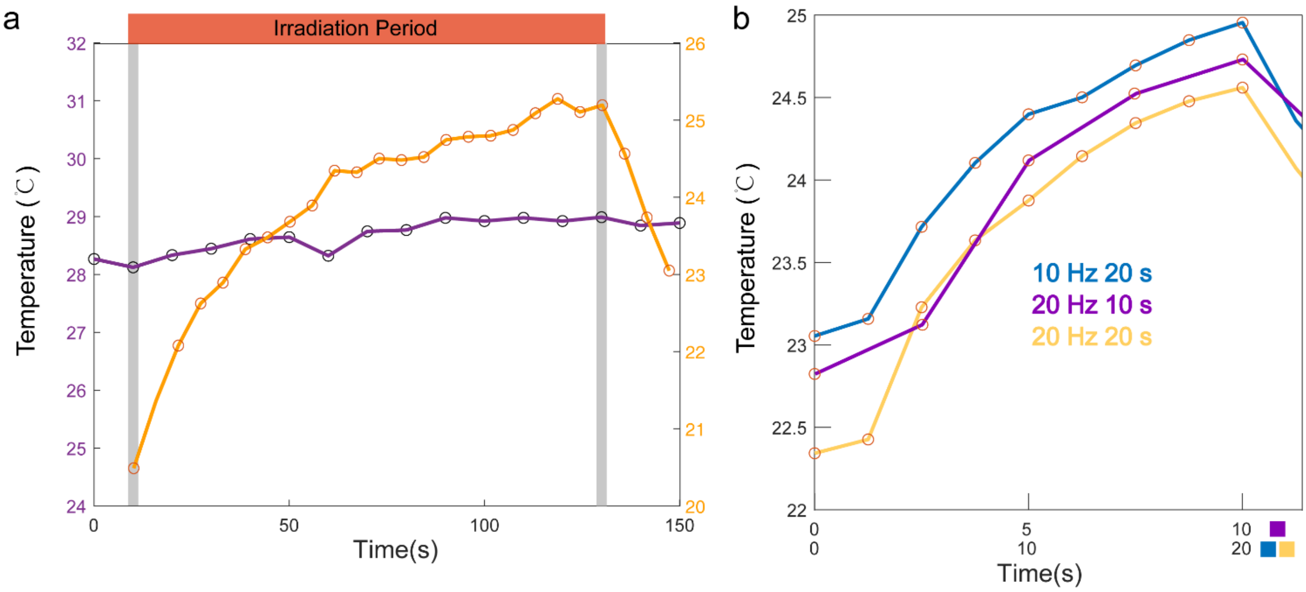

c

Power density (mw/mm²)			Frequency	Total duration(s)	Temperature rise (℃)
808 nm	980 nm	1532 nm			
59.1	38.8	0	5 Hz, 0.1 s on	120	~ 4.5
0.8	1.27	1.27	5 Hz, 0.1 s on	120	~ 1
0.8	3.74	1.43	10 Hz, 50 ms on	20	~ 2
1.5	2.75	1.59	20 Hz, 25 ms on	10	~ 2
0.8	2.75	1.59	20 Hz, 25 ms on	20	~ 2

Supplementary Fig. 30. Curves of temperature rises under different conditions. a, Purple line, the power densities of 808 nm, 980 nm and 1532 nm laser are 0.8 mW/mm^2, 1.27 mW/mm^2 and 1.27 mW/mm^2, respectively. Orange line, the power densities of 808 nm, 980 nm are 59.1 mW/mm^2, 38.8 mW/mm^2, respectively. Stimulation pattern: 5 Hz, 0.1 s on, 0.1 s off, 120 s in total. The red bar indicates time period of irradiation and the left and right gray column indicates the starting and ending frames of irradiation. **b,** Blue curve, 0.8 mW/mm^2 808 nm, 3.74 mW/mm^2 980 nm and 1.43 mW/mm^2 1532 nm laser, 50 ms on, 50 ms off, total duration 20 s. Purple curve, 1.5 mW/mm^2 808 nm, 2.75 mW/mm^2 980 nm and 1.59 mW/mm^2 1532 nm laser, 25 ms on, 25 ms off, total duration 10 s. Yellow curve, 0.8 mW/mm^2 808 nm, 2.75 mW/mm^2 980 nm and 1.59 mW/mm^2 1532 nm laser, 25 ms on, 25 ms off, total duration 20 s. **c,** Summary of used conditions and corresponding temperature rise. Source data are provided as a Source Data file.

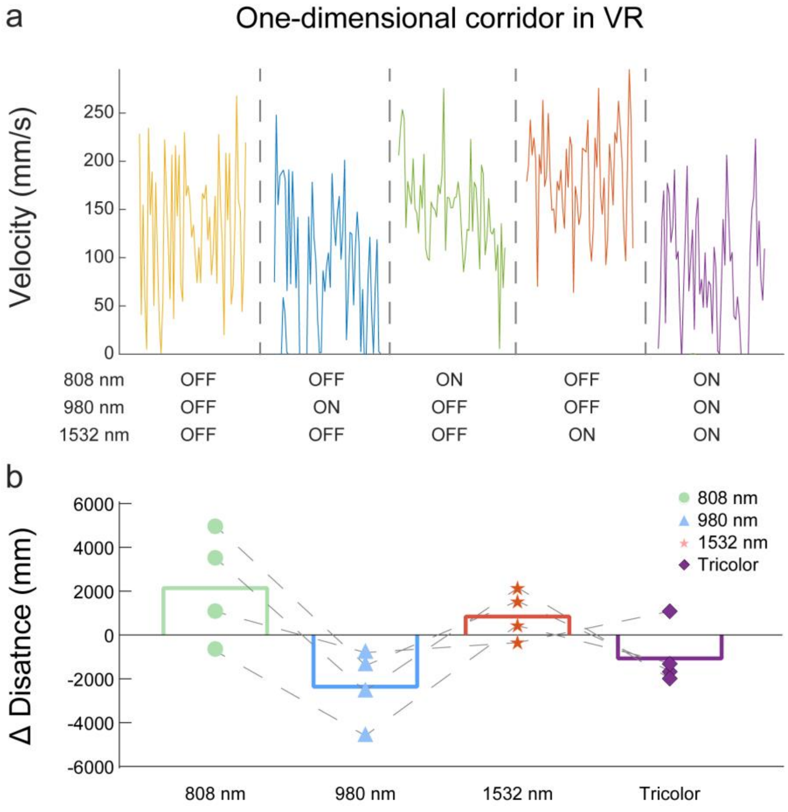

Supplementary Fig. 31. Transcranial optogenetic multi-color manipulation of mouse locomotion behavior using UCNPs. a, Running speed of example trials in one-dimensional corridor in VR system under 0.8 mW/mm^2 808 nm, 1.27 mW/mm^2 980 nm, 1.27 mW/mm^2 1532 nm and trichromatic NIR excitations (0.1 s on, 0.1 s off, 5 Hz, total duration 120 s). **b,** Delta running distances in one-dimensional corridor in VR system under 0.8 mW/mm^2 808 nm, 1.27 mW/mm^2 980 nm, 1.27 mW/mm^2 1532 nm and trichromatic NIR excitations (0.1 s on, 0.1 s off, 5 Hz, total duration 120 s) compared with control (no light stimulation) trials. (n = 4 mice, 3 trials for each mouse). Green circle, 808 nm excitation. Blue triangle, 980 nm excitation. Red star, 1532 nm excitation. Purple diamond, triple excitation. Source data are provided as a Source Data file.

Supplementary Table 1. Excitation condition and corresponding CIE points of trichromatic UCNPs.

Power Density (W/cm^{-2})			x	y	Point Number
980 nm	808 nm	1532 nm			
3.4410	0	–	0.15	0.11	（1）
3.4410	3.0914	–	0.24	0.33	（2）
3.4410	5.3374	–	0.24	0.38	（3）
3.4410	9.6308	–	0.24	0.43	（4）
3.4410	13.2671	–	0.24	0.47	（5）
–	9.0210	2.6432	0.26	0.67	（6）
–	9.0210	5.2355	0.37	0.59	（7）
–	9.0210	7.0792	0.45	0.52	（8）
–	9.0210	9.5187	0.51	0.46	（9）
–	9.0210	11.077	0.6	0.37	（10）
0	–	0.6009	0.69	0.3	（11）
0.7356	–	0.6009	0.57	0.31	（12）
1.3157	–	0.6009	0.45	0.31	（13）
1.6693	–	0.6009	0.3	0.19	（14）
2.0938	–	0.6009	0.23	0.13	（15）
3.6202	5.7210	11.0149	0.3 5	0.36	（16）

Supplementary Table2, Summary for power densities of NIR laser and visible laser in manuscript.

Figure	Stimulation pattern	Skull	Skin	Depth (μm)	Wavelength (nm)	Power density (mW/mm^2)	Converted light intensity(mW/mm^2)
Fig.4 g,h	1 s on– 19 s off	-	-	0	808	86.40	-
		-	-	0	980	89.10	-
		-	-	0	1532	76.80	-
		-	-	400	808	18.00	0.14
		-	-	400	980	24.39	0.44
		-	-	400	1532	20.21	0.014
Fig.5d	1s on– 19s off	√	√	0	530	0.07	-
		√	√	0	470	0.44	-
		√	√	0	808	86.40	-
		√	√	0	980	89.10	-
		√	√	400	530	0.0010	-
		√	√	400	470	0.0047	-
		√	√	400	808	7.08	0.06
		√	√	400	980	12.18	0.23
Fig.5g	1s on-19 s off	-	-	0	808	86.40	
		-	-	0	980	61.90	
		-	-	400	808	18.00	0.14
		-	-	400	980	16.95	0.30
Fig.4e	1s on– 9 s off	-	-	0	530	0.07	-
		-	-	0	470	0.44	-
		-	-	0	650	0.83	-
		-	-	400	530	0.0039	-
		-	-	400	470	0.014	-
		-	-	400	650	0.13	-
Fig.5 e,f i,j	0.1 s on– 0.1 s off	√	-	0	808	59.10	-
		√	-	0	980	38.90	-
		√	-	400	808	10.38	0.083
		√	-	400	980	9.02	0.16

Supplementary Table3, Summary for statistical details in manuscript.

Figure	Group	p value	n	Statistical methods
Fig. S26d middle	808 nm vs 980 nm	$p < 0.001$	6 neurons	one-way repeated measures ANOVA with Tukey's multiple comparison test
Fig. S26d middle	808 nm vs 1532 nm	$p < 0.001$	6 neurons	
Fig. S26d top	980 nm vs 808 nm	$p < 0.001$	9 neurons	
Fig. S26d top	980 nm vs 1532 nm	$p < 0.001$	9 neurons	
Fig. S26d bottom	1532 nm vs 808 nm	$p = 0.0053$	5 neurons	
Fig. S26d bottom	1532 nm vs 980 nm	$p = 0.0174$	5 neurons	
Fig. 5d top	808 nm vs 530 nm, depth at 0.4mm with skull	$p < 0.001$	4 neurons	
Fig. 5d bottom	980 nm vs 470 nm, depth at 0.4 mm with skull	$p < 0.001$	6 neurons	
Fig. 5d bottom	980 nm vs 470 nm, depth at 0.7 mm with skull	$p = 0.006$	6 neurons	
Fig. 5e bottom	808 nm off, 980 nm off vs 808 nm on, 980 nm off	$p < 0.001$	8 mice	
Fig. 5e bottom	808 nm off, 980 nm off vs 808 nm off, 980 nm on	$p = 0.033$	8 mice	
Fig. 5e bottom	808 nm on, 980 nm off vs 808 nm on, 980 nm on	$p = 0.002$	8 mice	
Fig. 5f bottom	808 nm off, 980 nm off vs 808 nm on, 980 nm off	$p = 0.0243$	8 mice	
Fig. 5f bottom	808 nm off, 980 nm off vs 808 nm off, 980 nm on	$p = 0.0399$	8 mice	
Fig. 5f bottom	808 nm on, 980 nm off vs 808 nm off, 980 nm on	$p = 0.0135$	8 mice	
Fig. 5f bottom	808 nm on, 980 nm off vs 808 nm on, 980 nm on	$p = 0.0471$	8 mice	

Figure	Power Density(mW/mm²)				p value	n (mice)	Statistical methods
Fig. 5i	808 nm<br>980 nm	59.1<br>4.9	VS	0<br>38.9	p = 0.002	6	
Fig. 5i	808 nm<br>980 nm	59.1<br>4.9	VS	59.1<br>38.9	p = 0.0018	6	
Fig. 5i	808 nm<br>980 nm	59.1<br>0	VS	0<br>38.9	p = 0.004	6	
Fig. 5i	808 nm<br>980 nm	59.1<br>0	VS	59.1<br>38.9	p = 0.033	6	
Fig. 5i	808 nm<br>980 nm	59.1<br>8.4	VS	0<br>38.9	p = 0.09	6	
Fig. 5i	808 nm<br>980 nm	59.1<br>14.1	VS	0<br>38.9	p = 0.01	6	
Fig. 5i	808 nm<br>980 nm	59.1<br>28.4	VS	0<br>38.9	p = 0.04	6	two-way repeated measures ANOVA with Tukey's multiple comparison test
Fig. 5j	808 nm<br>980 nm	0<br>0	VS	59.1<br>4.9	p = 0.0445	6	
Fig. 5j	808 nm<br>980 nm	59.1<br>0	VS	0<br>38.9	p = 0.0465	6	
Fig. 5j	808 nm<br>980 nm	59.1<br>4.9	VS	59.1<br>38.9	p = 0.0024	6	
Fig. 5j	808 nm<br>980 nm	59.1<br>4.9	VS	0<br>38.9	p < 0.001	6	
Fig. 5j	808 nm<br>980 nm	59.1<br>8.4	VS	59.1<br>38.9	p = 0.0121	6	
Fig. 5j	808 nm<br>980 nm	59.1<br>8.4	VS	0<br>38.9	p < 0.001	6	
Fig. 5j	808 nm<br>980 nm	59.1<br>14.1	VS	0<br>38.9	p = 0.0077	6	

Figure	Neurons Prefered Visible Light	Activate Wavelength	p value	n	Statistical methods
Fig.4e	Blue-light	470nm VS 530nm	P < 0.001	7 neurons	one-way repeated measures ANOVA with Tukey's multiple comparison test
Fig.4e	Blue-light	470nm VS 630nm	P < 0.001	7 neurons	
Fig.4e	Green-light	530nm VS 470nm	P = 0.0115	8 neurons	
Fig.4e	Green-light	530nm VS 630nm	P = 0.0073	8 neurons	
Fig.4e	Red-light	630nm VS 470nm	P = 0.0361	4 neurons	
Fig.4e	Red-light	630nm VS 530nm	P = 0.0415	4 neurons	

Reference

1. Johnson NJJ, Wendy O, Stanisz GJ, Scott PR, Van VFCJM. Size-Tunable, Ultrasmall $NaGdF_4$ Nanoparticles: Insights into their T1 MRI Contrast Enhancement. *Cheminform* 2011, **42**(44)**:** 3714-3722.

2. Li X, Shen D, Yang J, Yao C, Che R, Zhang F*, et al.* Successive Layer-by-Layer Strategy for Multi-Shell Epitaxial Growth: Shell Thickness and Doping Position Dependence in Upconverting Optical Properties. *Chem Mater* 2013, **25**(1)**:** 106-112.

3. Hong G, Antaris AL, Dai H. Near-infrared fluorophores for biomedical imaging. *Nat Biomed Eng* 2017, **1**(1)**:** 0010.

4. Zhao J, Chen B, Wang F. Shedding Light on the Role of Misfit Strain in Controlling Core-Shell Nanocrystals. *Adv Mater* 2020, **32**(46)**:** e2004142.

5. Kastella KG, Fox JR. The Dynamic Response of Brain Temperature to Localized Heating. *Biophys J* 1971, **11**(6)**:** 521-539.

6. Jafari F, Higgins PD. Thermal modeling in cylindrical coordinates using effective conductivity. *IEEE Trans Ultrason Ferroelectr Freq Control* 1989, **36**(2)**:** 191-196.
