# Supplementary material for: Near-infrared manipulation of multiple neuronal populations via trichromatic upconversion
Source: Nat Commun. 2021 Sep 27;12:5662. doi: 10.1038/s41467-021-25993-7 (PMC8476604; doi:10.1038/s41467-021-25993-7)
Supplement: Supplementary file 2 — Reporting Summary [file 41467_2021_25993_MOESM2_ESM.pdf]

nature portfolio

Corresponding author(s): Fan Zhang, Jiayi Zhang, Xiaomin Li

Last updated by author(s): Aug 23, 2021

Reporting Summary

Nature Portfolio wishes to improve the reproducibility of the work that we publish. This form provides structure for consistency and transparency in reporting. For further information on Nature Portfolio policies, see our Editorial Policies and the Editorial Policy Checklist.

Statistics

For all statistical analyses, confirm that the following items are present in the figure legend, table legend, main text, or Methods section.

n/a	Confirmed	
☐	☒	The exact sample size (*n*) for each experimental group/condition, given as a discrete number and unit of measurement
☐	☒	A statement on whether measurements were taken from distinct samples or whether the same sample was measured repeatedly
☐	☒	The statistical test(s) used AND whether they are one- or two-sided<br>*Only common tests should be described solely by name; describe more complex techniques in the Methods section.*
☒	☐	A description of all covariates tested
☐	☒	A description of any assumptions or corrections, such as tests of normality and adjustment for multiple comparisons
☐	☒	A full description of the statistical parameters including central tendency (e.g. means) or other basic estimates (e.g. regression coefficient) AND variation (e.g. standard deviation) or associated estimates of uncertainty (e.g. confidence intervals)
☐	☒	For null hypothesis testing, the test statistic (e.g. *F*, *t*, *r*) with confidence intervals, effect sizes, degrees of freedom and *P* value noted<br>*Give P values as exact values whenever suitable.*
☒	☐	For Bayesian analysis, information on the choice of priors and Markov chain Monte Carlo settings
☒	☐	For hierarchical and complex designs, identification of the appropriate level for tests and full reporting of outcomes
☒	☐	Estimates of effect sizes (e.g. Cohen's *d*, Pearson's *r*), indicating how they were calculated

Our web collection on statistics for biologists contains articles on many of the points above.

Software and code

Policy information about availability of computer code

Data collection	A custom script was used to control the laser powers with Cyberlease scanning software for the propose-built synchronous scanning system and full-color images. In vivo electrophysiology data was collected by apollo software embedded in the Bio-Signal Technologies instrument (V4.4.10). Pulse stimulation of lasers was controlled by Arduino Uno and the data was collected by Clampex 10.5 in patch clamp experiments. In behavioral experiments, data was collected by PhenoSoft Control software (version 1.0.5693.30358). Temperature data was collected by FLIR IR Monitor (version 1.6.0.0).
Data analysis	Imaging analysis: MATLABR2015a;<br>Statistic Analysis: Sigmaplot (SYSTAT, USA), OriginPro8, GraphPad Prism8;<br>In vivo electrophysiology data analysis: CED Spike2, Plexon offline sorter, MATLAB R2015a;<br>Numerical Simulation:MATLAB R2015a, Python3.7

For manuscripts utilizing custom algorithms or software that are central to the research but not yet described in published literature, software must be made available to editors and reviewers. We strongly encourage code deposition in a community repository (e.g. GitHub). See the Nature Portfolio guidelines for submitting code & software for further information.

Data

Policy information about availability of data

All manuscripts must include a data availability statement. This statement should provide the following information, where applicable:

- Accession codes, unique identifiers, or web links for publicly available datasets
- A description of any restrictions on data availability
- For clinical datasets or third party data, please ensure that the statement adheres to our policy

The source data underlying figures are provided as public opening Source Data file. Data supporting the findings of this study are available within the article and the associated Supplementary Information. Any other data are available from the corresponding authors upon reasonable request.

Field-specific reporting

Please select the one below that is the best fit for your research. If you are not sure, read the appropriate sections before making your selection.

- [x] Life sciences
- [ ] Behavioural & social sciences
- [ ] Ecological, evolutionary & environmental sciences

For a reference copy of the document with all sections, see nature.com/documents/nr-reporting-summary-flat.pdf

Life sciences study design

All studies must disclose on these points even when the disclosure is negative.

Sample size	No sample size calculations were performed. Sample sizes were determined by the number of biological replicates which are necessary for ensuring statistical significant, meeting the criteria of the National Institutes of Health Guide for the Care and Use of Laboratory Animals, The sample size of each experiment is provided in the corresponding figure captions.
Data exclusions	No data were excluded from the analyses.
Replication	Animal experiments were performed on biological replicates following identical procedures to verify the reproducibility of the experimental findings in 6 months and all attempts at replication were successful. Animal behavioral experiments, in vivo electrophysiological experiments, immunohistochemistry and patch clamp experiments were repeated at least with 2 independent biological repeats.
Randomization	The mice used in experiments were randomly allocated into the positive and control groups.
Blinding	Investigators were not blinded to group allocation during data collection and analysis, since all data were collected and assembled by instrument software without interference. Investigators performed the same procedure to data process.

Reporting for specific materials, systems and methods

We require information from authors about some types of materials, experimental systems and methods used in many studies. Here, indicate whether each material, system or method listed is relevant to your study. If you are not sure if a list item applies to your research, read the appropriate section before selecting a response.

Materials & experimental systems

n/a	Involved in the study	
☐	☒	Antibodies
☒	☐	Eukaryotic cell lines
☒	☐	Palaeontology and archaeology
☐	☒	Animals and other organisms
☒	☐	Human research participants
☒	☐	Clinical data
☒	☐	Dual use research of concern

Methods

n/a	Involved in the study	
☒	☐	ChIP-seq
☒	☐	Flow cytometry
☒	☐	MRI-based neuroimaging

Antibodies

Antibodies used	1.Anti-GFP antibody, Cat# GFP-1020, 1:800, Avēs Lab Inc, US<br>2.Anti-mCherry antibody, Cat# AB0081-200, 1:2000, SICGEN, Portugal<br>3.Anti-PV antibody, Cat# PV 25, 1:800, Swant Inc, Swiss<br>4.Anti-Iba1 antibody, Cat# ab5076, 1:500, Abcam, UK<br>5.Anti-GFAP antibody, Cat# G6171, 1:1000, Sigma, US<br>6.Donkey anti chicken 488 secondary antibody, Cat# 705-545-155, 1:300, Jackson ImmunoResearch, US

	7.Donkey anti goat 594 secondary antibody, Cat# 705-585-003, 1:200, Jackson ImmunoResearch, US<br>8.Donkey anti rabbit 594 secondary antibody, Cat# 711-585-152,1:200, Jackson ImmunoResearch, US<br>9.Donkey anti rabbit 647 secondary antibody, Cat# 711-605-152, 1:200, Jackson ImmunoResearch, US<br>10.Donkey anti mouse 488 secondary antibody, Cat#715-545-150, 1:200, Jackson ImmunoResearch, US.
Validation	Validation details of the primary antibodies are available on the manufacturers' websites and the related references:<br>1.GFP, Cat#GFP-1020 antibody, http://www.aveslab.com/products/epitope-tag-and-gfp-antibodies/anti-gfp-green-fluorescent-proteinantibodies-2/, Diao Y et al (pubmed id: 28334242)<br>2.mCherrry antibody, Cat# AB0081-200, http://www.sicgen.pt/product/mcherry-polyclonal-antibody_1_27, Saito K et al (pubmed id:29458175)<br>3.PV, Cat# PV 25 antibody, https://www.swant.com/?p=products&c=1.3, Filice F et al (pubmed id: 28675430)<br>4.GFAP, Cat# G6171, https://www.sigmaaldrich.cn/CN/zh/product/sigma/g6171?context=product, Zayas-Santiago A., Agte S., et al. (pubmed id: 24831221)<br>5.Iba1, Cat# ab5076, https://www.abcam.cn/iba1-antibody-ab5076.html , Keane L et al.(pubmed id: 33108356).

Animals and other organisms

Policy information about studies involving animals; ARRIVE guidelines recommended for reporting animal research

Laboratory animals	Six-week old C57BL/6J male mice were purchased from Shanghai Laboratory Animal Center, Chinese Academy of Sciences. Male PV-Cre mice (Jackson lab, US. Stock #008069) and SOM-Flp mice (Jackson lab, US. Stock #031629) aged from six-week to fourteen-week were used in experiments.
Wild animals	The study do not involved wild animals.
Field-collected samples	No filed-collected samples were used in the study.
Ethics oversight	All protocols for animal experiments were in strict accordance with the National Institutes of Health Guide for the Care and Use of Laboratory Animals, and approved by Animal Care and Use Committee of Fudan University.

Note that full information on the approval of the study protocol must also be provided in the manuscript.
