# Supplementary material for: Near-infrared manipulation of multiple neuronal populations via trichromatic upconversion
Source: Nat Commun. 2021 Sep 27;12:5662. doi: 10.1038/s41467-021-25993-7 (PMC8476604; doi:10.1038/s41467-021-25993-7)
Supplement: Supplementary file 3 — Description of Additional Supplementary Files [file 41467_2021_25993_MOESM3_ESM.pdf]

Title: Supplementary Movie 1:
Description: Trichromatic UCNPs for orthogonal RGB emission

Title: Supplementary Movie 2:
Description: NIR dual-color control of mice motor function
